# Supplementary material for: A Brain Morphometry Study with Across-Site Harmonization Using a ComBat-Generalized Additive Model in Children and Adolescents
Source: Diagnostics (Basel). 2023 Aug 27;13(17):2774. doi: 10.3390/diagnostics13172774 (PMC10487204; doi:10.3390/diagnostics13172774)
Supplement: Supplementary file 1 [file diagnostics-13-02774-s001.zip › BASH-NC Figure S1.pdf]

Figure S1: ComBat-GAM harmonization Scatter plot of regional brain volumes for scan age and scan site pre- (left panel) and after ComBat-GAM harmonization (right panel).

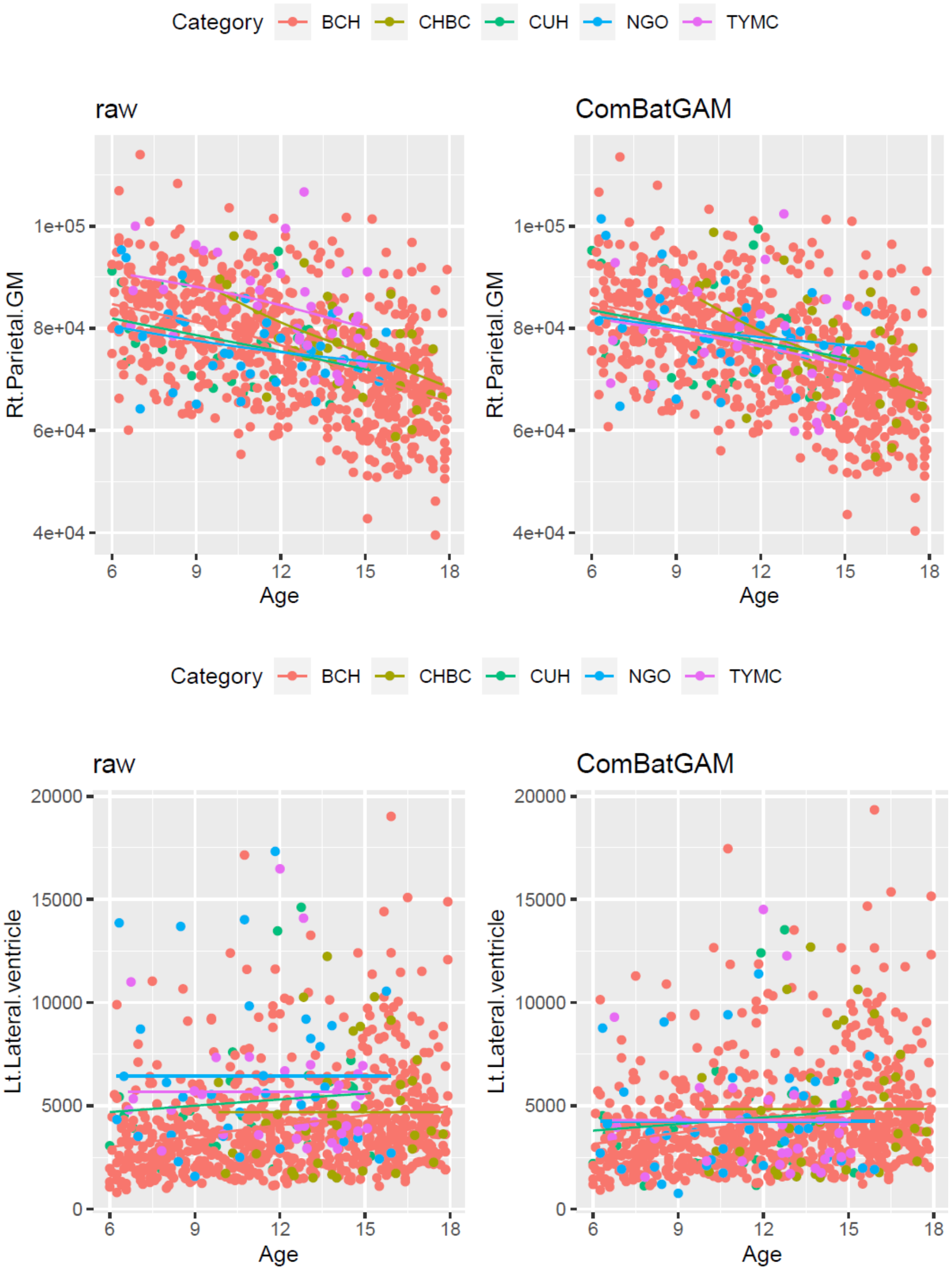

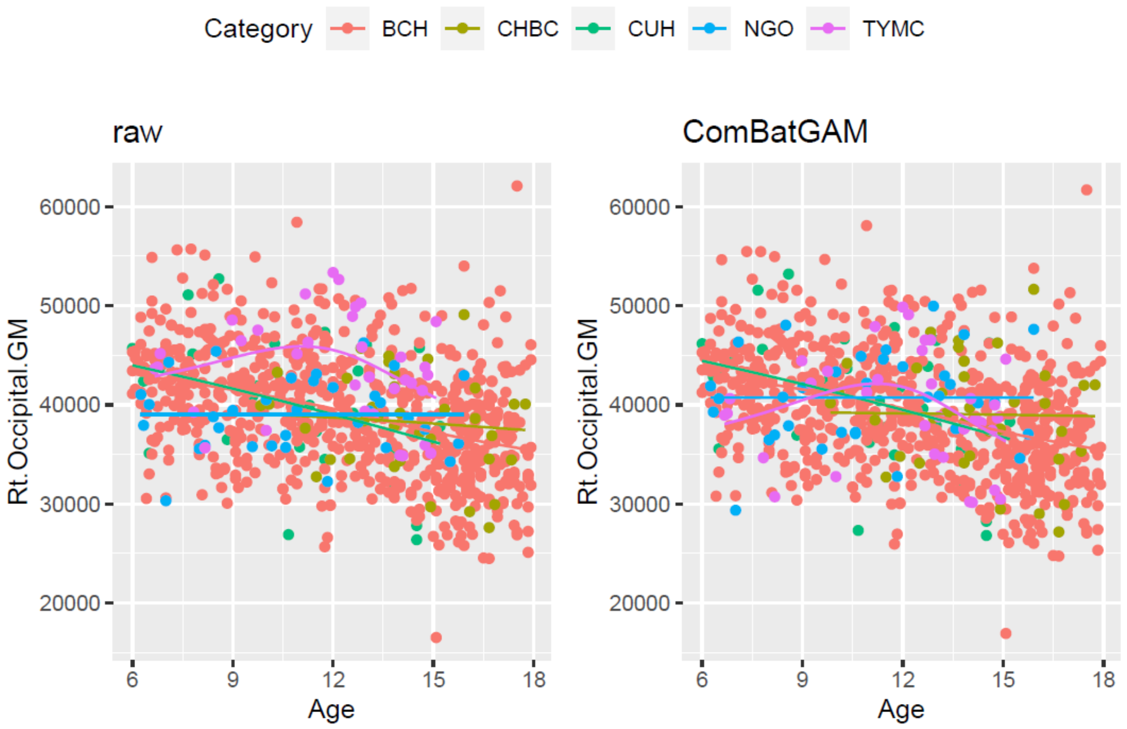
Category BCH CHBC CUH NGO TYMC
raw
ComBatGAM
Rt.Occipital.GM
60000
50000
40000
30000
20000
6 9 12 15 18
Age

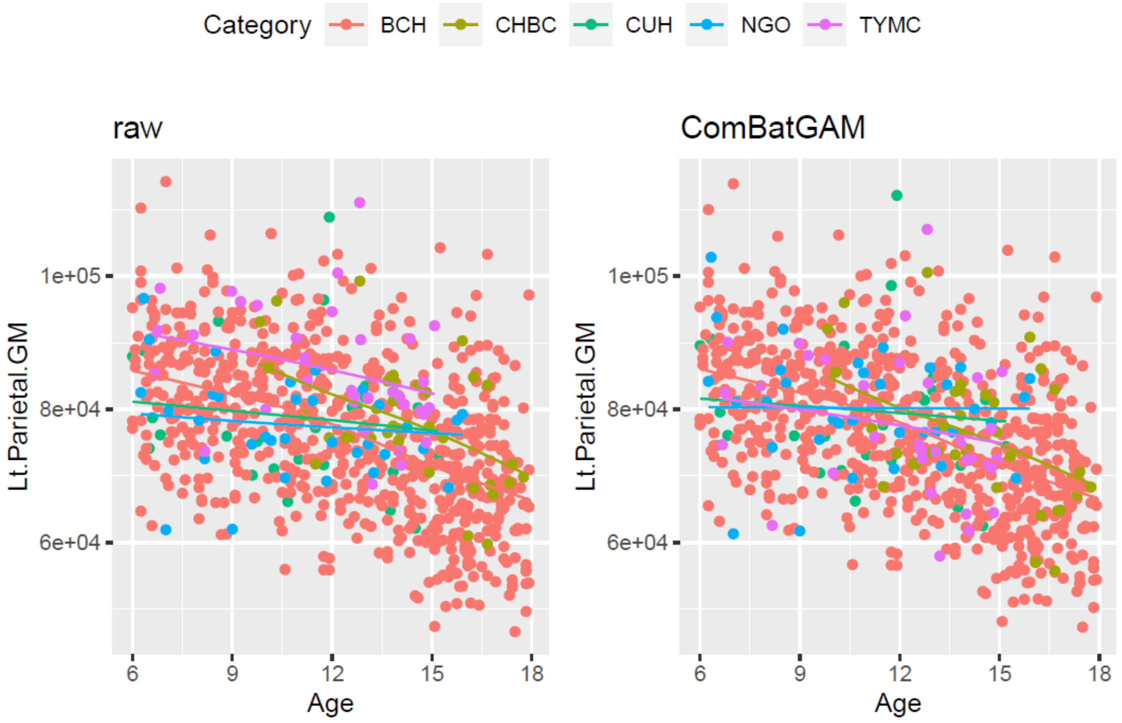
Category BCH CHBC CUH NGO TYMC
raw
ComBatGAM
Lt.Parietal.GM
1e+05
8e+04
6e+04
6 9 12 15 18
Age

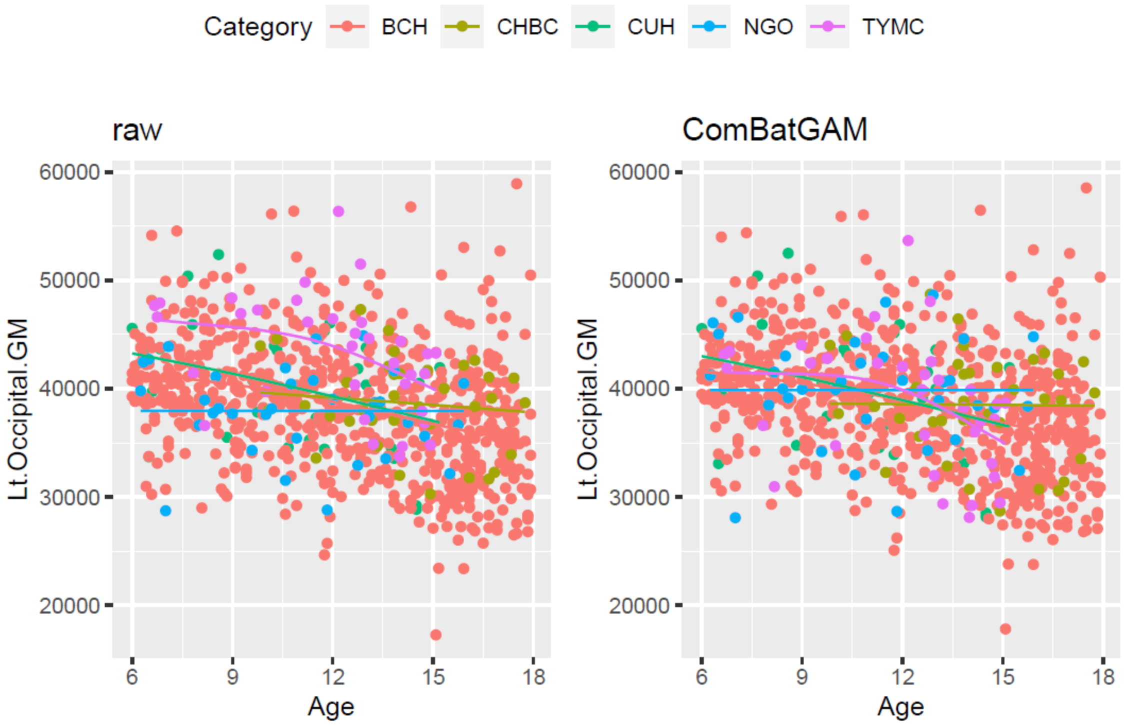
Category
BCH
CHBC
CUH
NGO
TYMC
raw
ComBatGAM
Lt.Occipital.GM
Age
60000
50000
40000
30000
20000
6
9
12
15
18

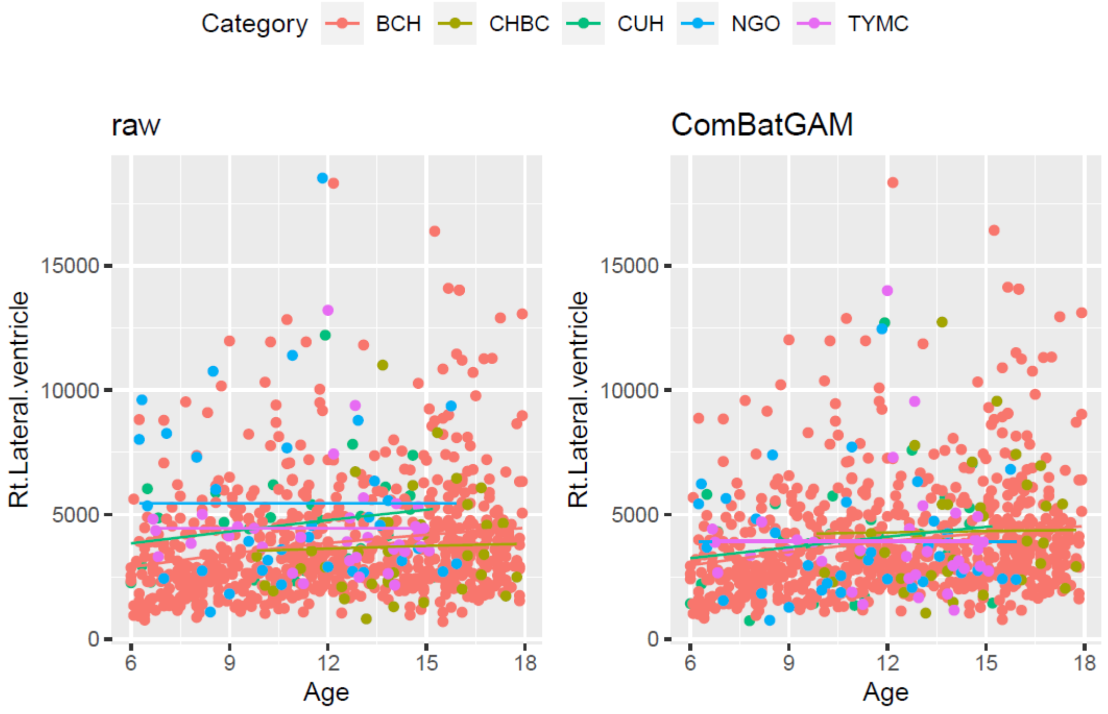
Category
BCH
CHBC
CUH
NGO
TYMC
raw
ComBatGAM
Rt.Lateral.ventricle
Age
15000
10000
5000
0
6
9
12
15
18

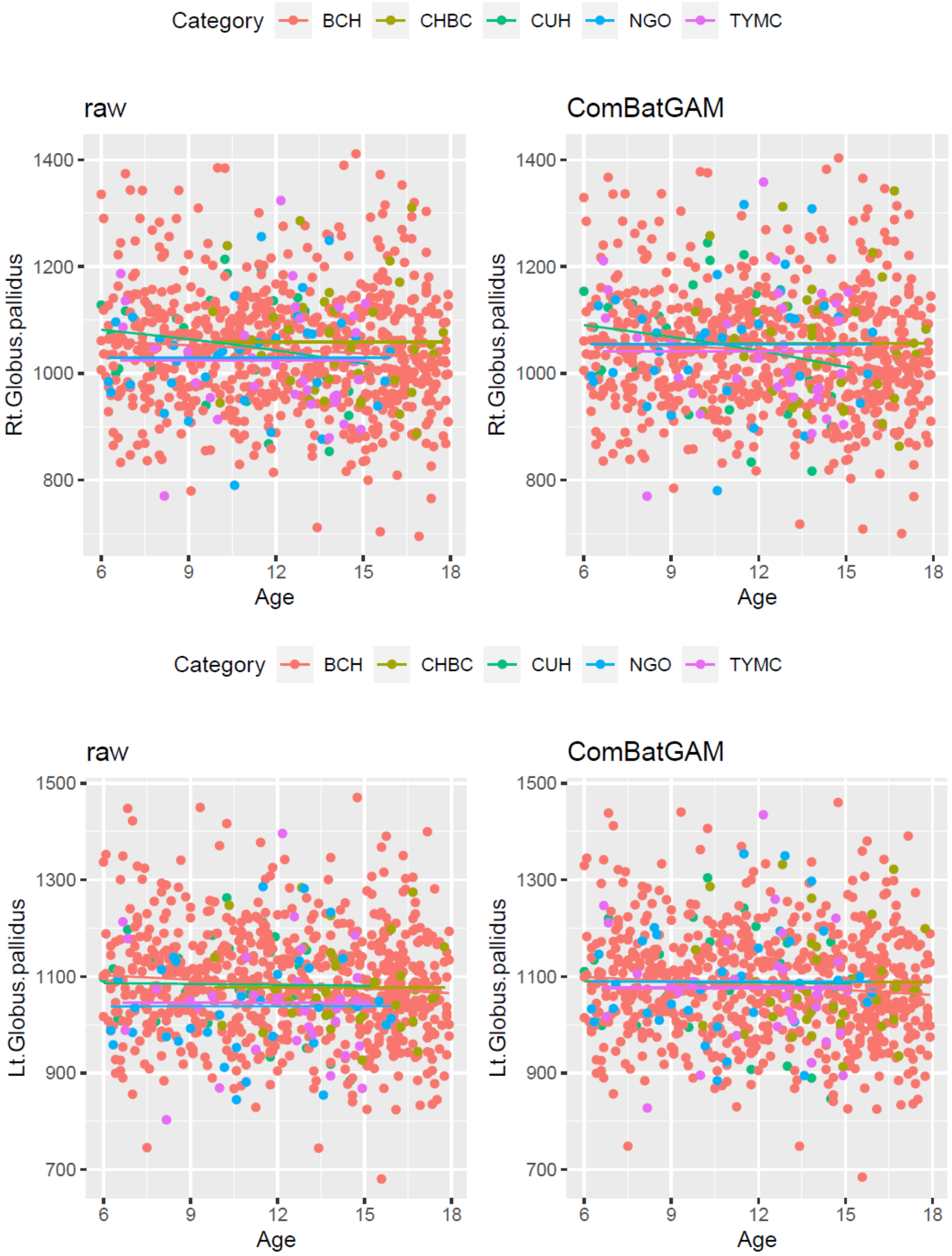
Category BCH CHBC CUH NGO TYMC
raw
ComBatGAM
Rt.Globus.pallidus
1400
1200
1000
800
Age
6
9
12
15
18
Category BCH CHBC CUH NGO TYMC
raw
ComBatGAM
Lt.Globus.pallidus
1500
1300
1100
900
700
Age
6
9
12
15
18

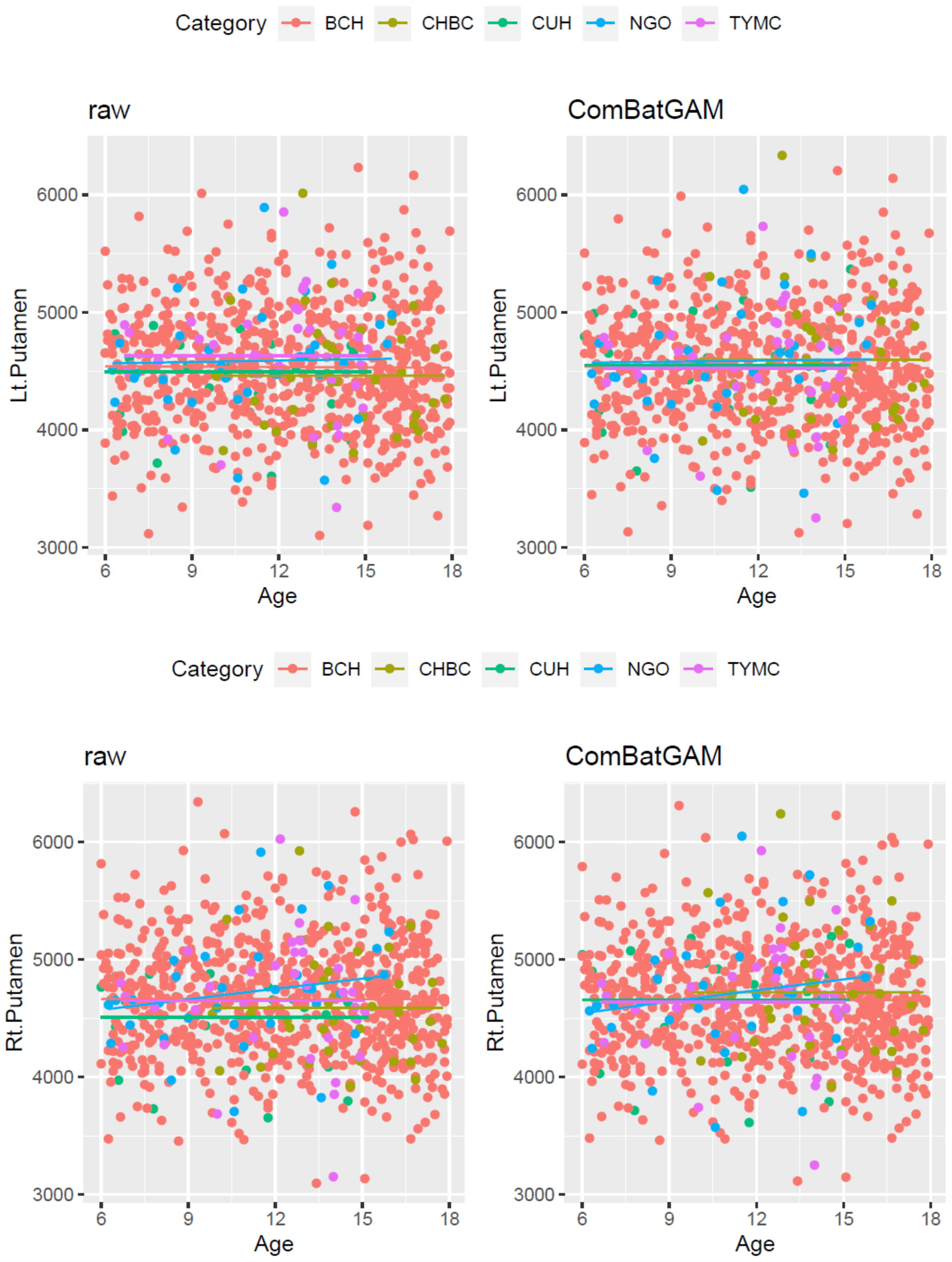
Category
BCH
CHBC
CUH
NGO
TYMC
raw
ComBatGAM
Lt.Putamen
3000
4000
5000
6000
6
9
12
15
18
Age
Category
BCH
CHBC
CUH
NGO
TYMC
raw
ComBatGAM
Rt.Putamen
3000
4000
5000
6000
6
9
12
15
18
Age

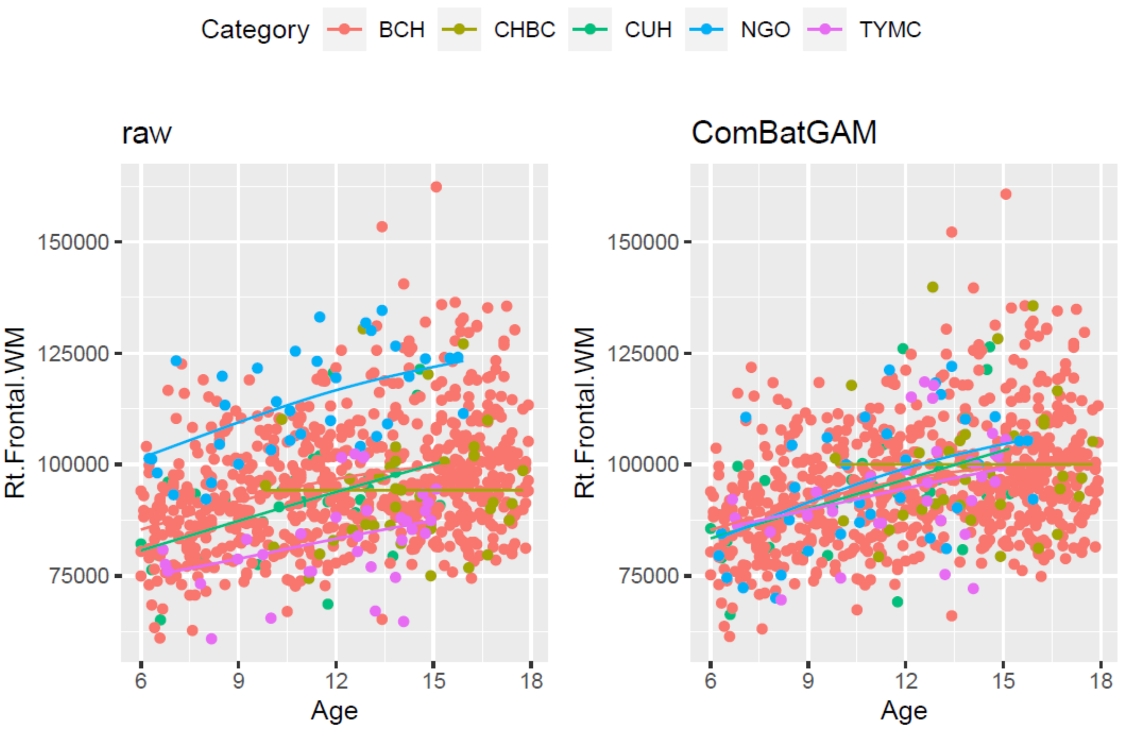
Category
BCH
CHBC
CUH
NGO
TYMC
raw
ComBatGAM
Rt.Frontal.WM
150000
125000
100000
75000
6
9
12
15
18
Age

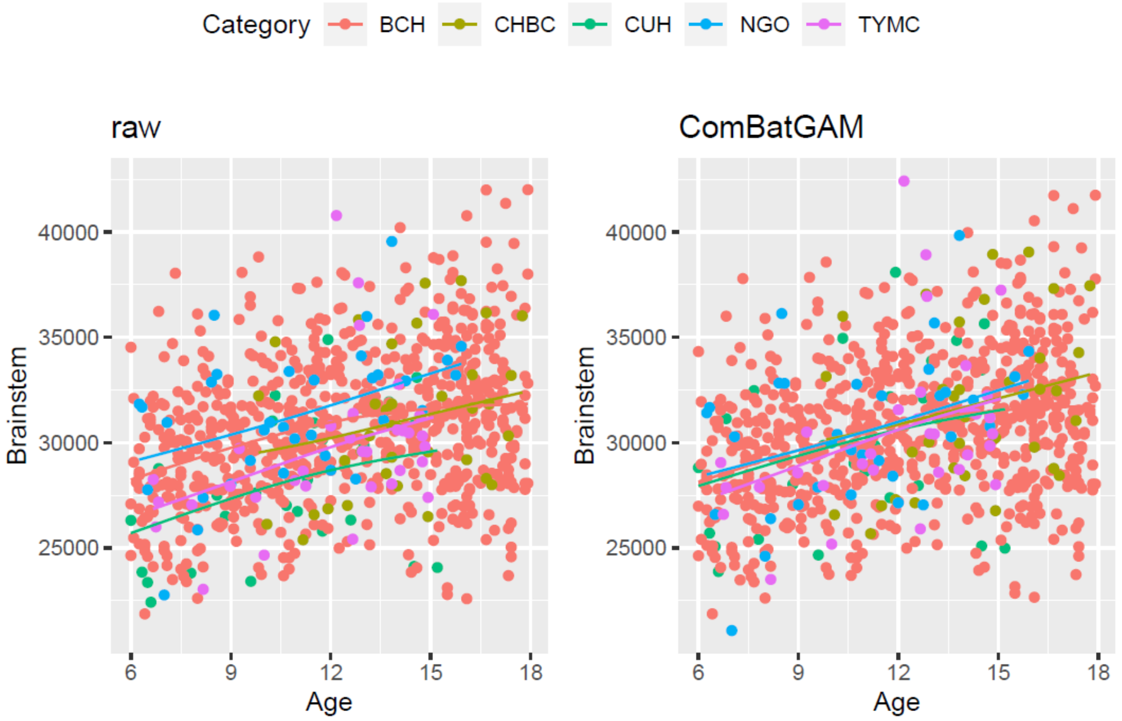
Category
BCH
CHBC
CUH
NGO
TYMC
raw
ComBatGAM
Brainstem
40000
35000
30000
25000
6
9
12
15
18
Age

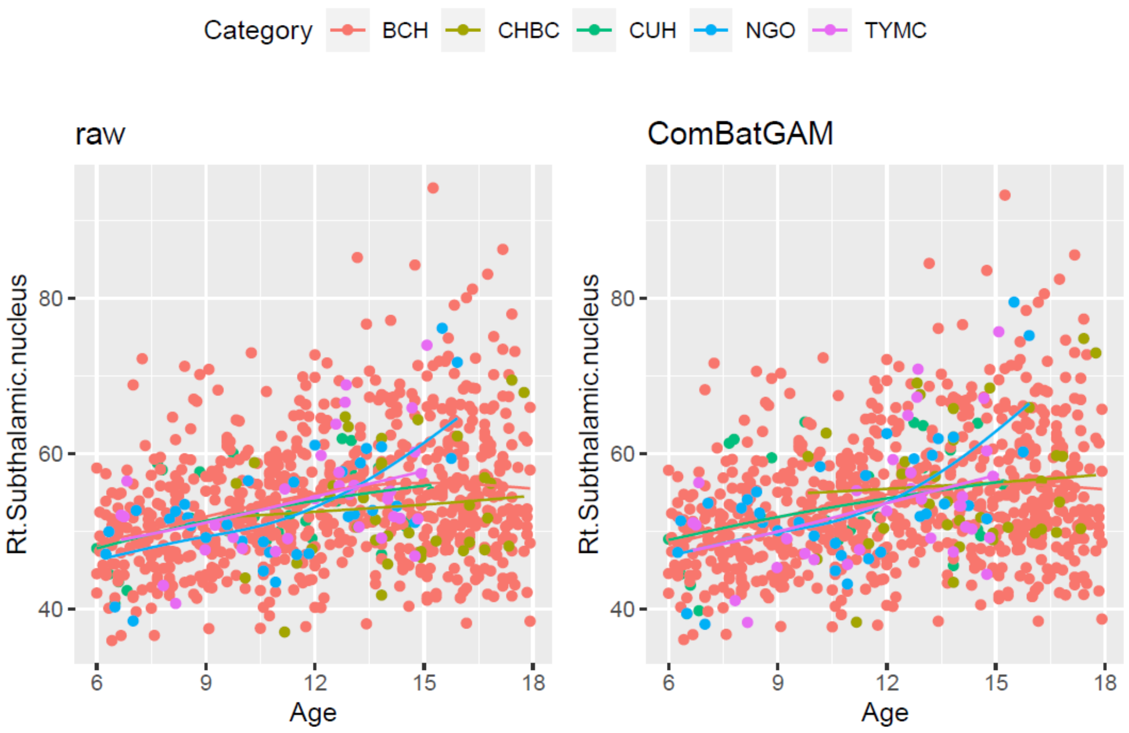
Category
BCH
CHBC
CUH
NGO
TYMC
raw
ComBatGAM
Rt.Subthalamic.nucleus
80
60
40
6
9
12
15
18
Age

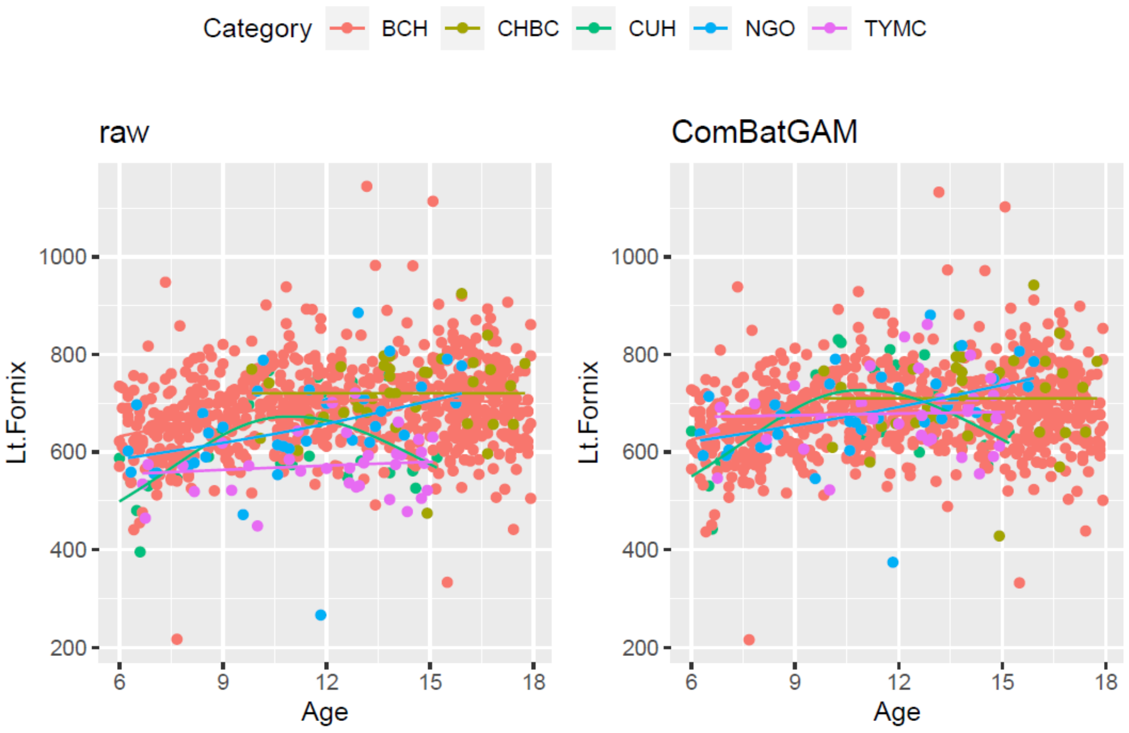
Category
BCH
CHBC
CUH
NGO
TYMC
raw
ComBatGAM
Lt.Fornix
1000
800
600
400
200
6
9
12
15
18
Age

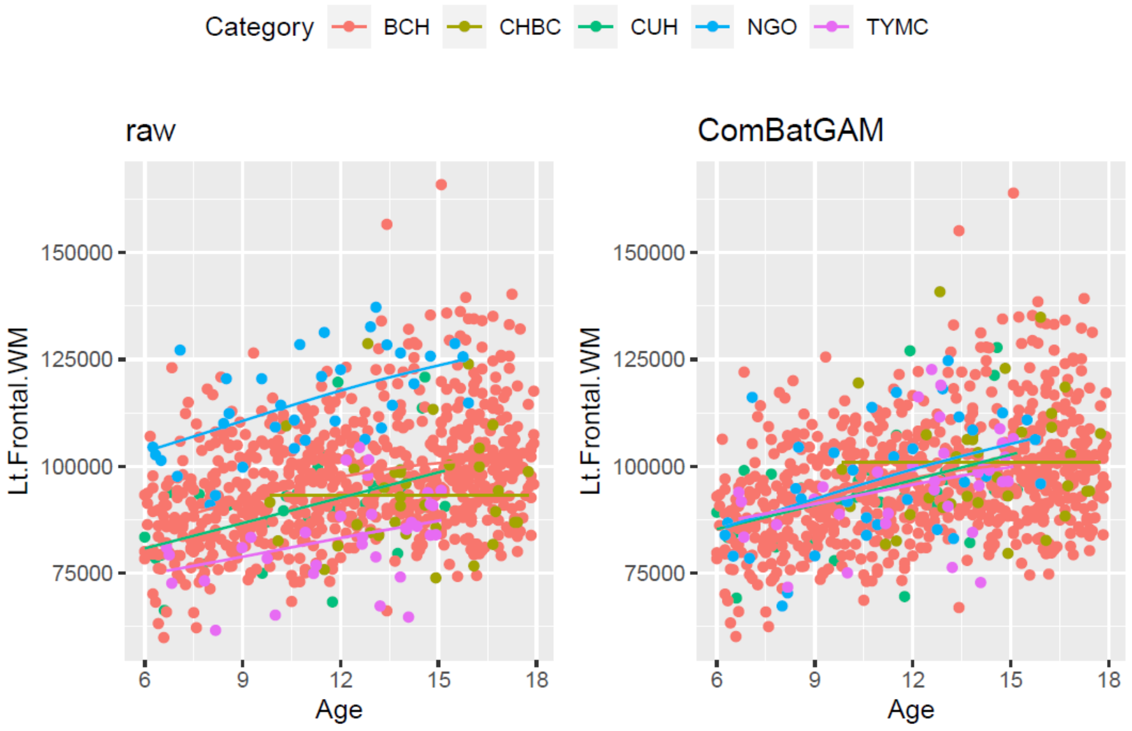
Category
BCH
CHBC
CUH
NGO
TYMC
raw
ComBatGAM
Lt.Frontal.WM
150000
125000
100000
75000
6
9
12
15
18
Age

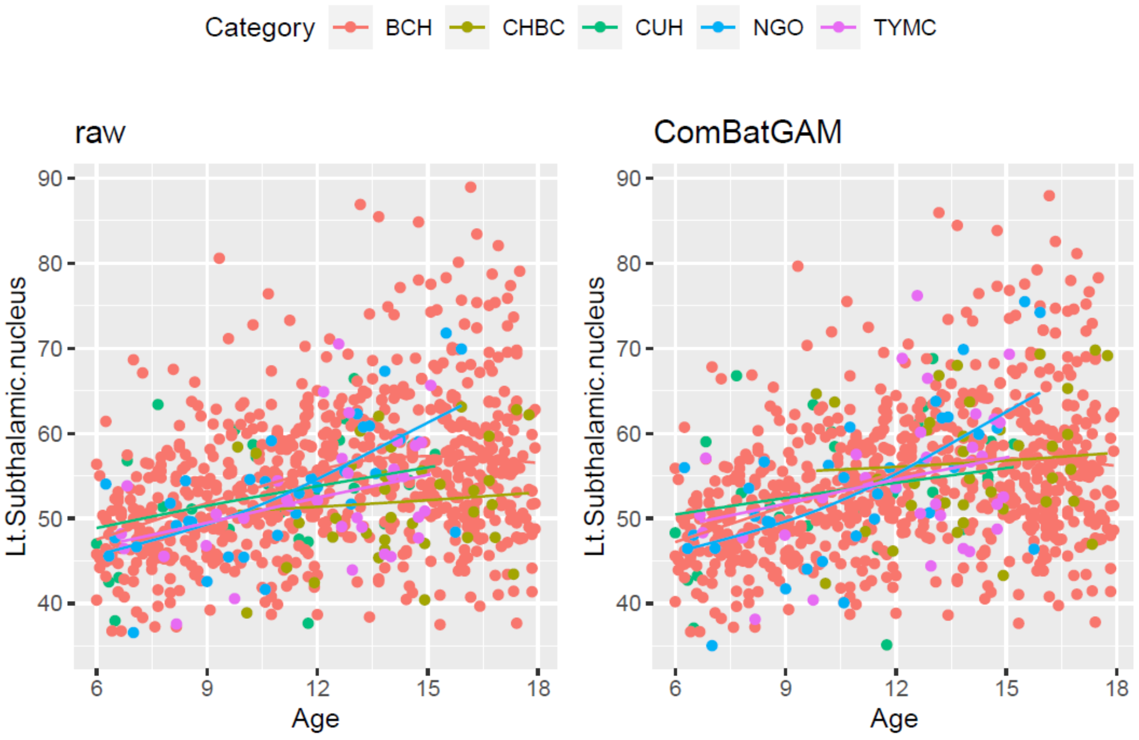
Category
BCH
CHBC
CUH
NGO
TYMC
raw
ComBatGAM
Lt.Subthalamic.nucleus
90
80
70
60
50
40
6
9
12
15
18
Age

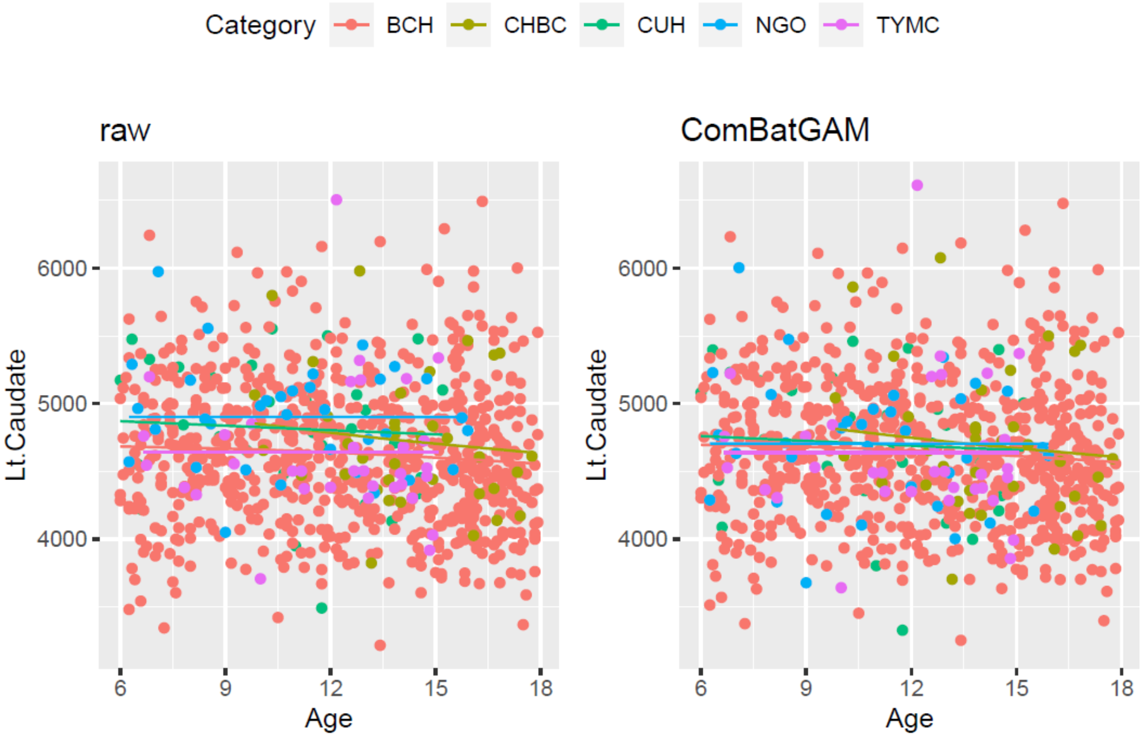
Category
BCH
CHBC
CUH
NGO
TYMC
raw
ComBatGAM
Lt.Caudate
6000
5000
4000
6
9
12
15
18
Age

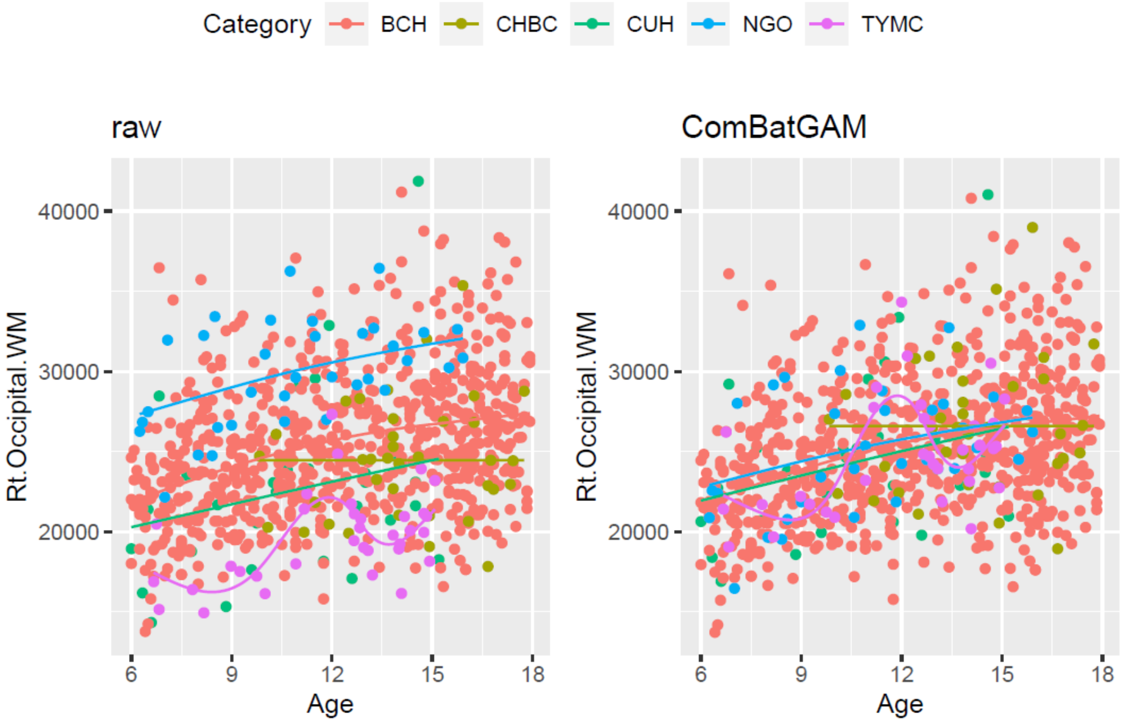
Category
BCH
CHBC
CUH
NGO
TYMC
raw
ComBatGAM
Rt.Occipital.WM
40000
30000
20000
6
9
12
15
18
Age

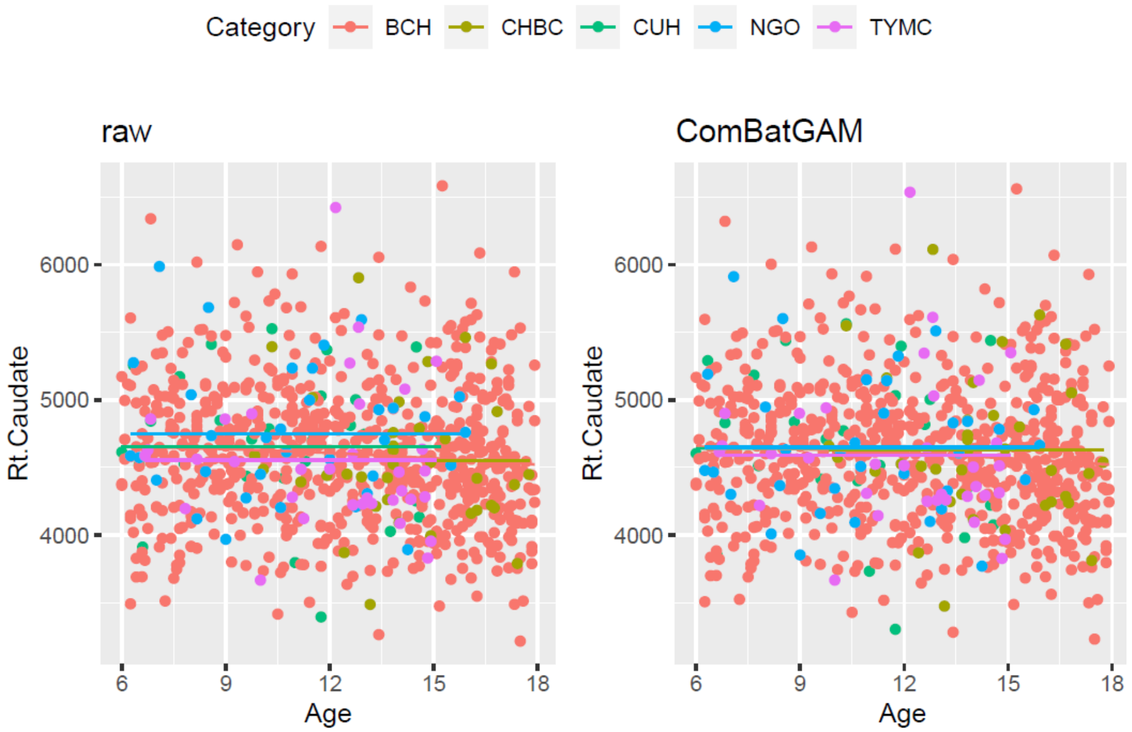
Category
BCH
CHBC
CUH
NGO
TYMC
raw
ComBatGAM
Rt.Caudate
6000
5000
4000
6
9
12
15
18
Age

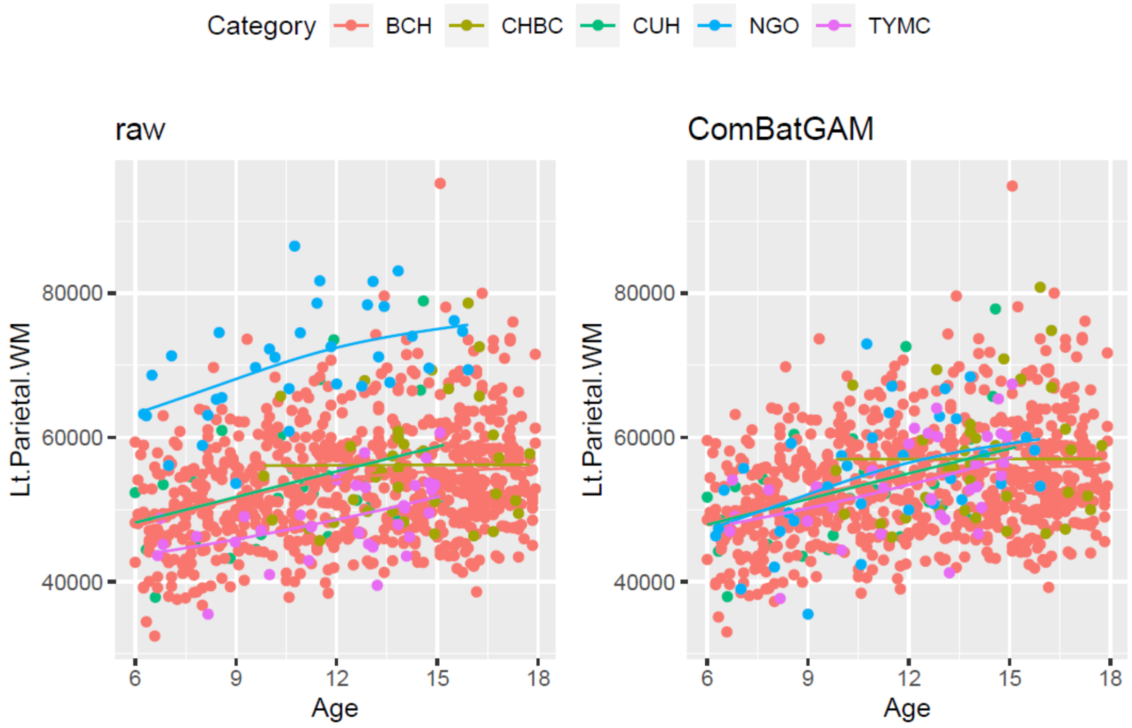
Category
BCH
CHBC
CUH
NGO
TYMC
raw
ComBatGAM
Lt.Parietal.WM
80000
60000
40000
6
9
12
15
18
Age

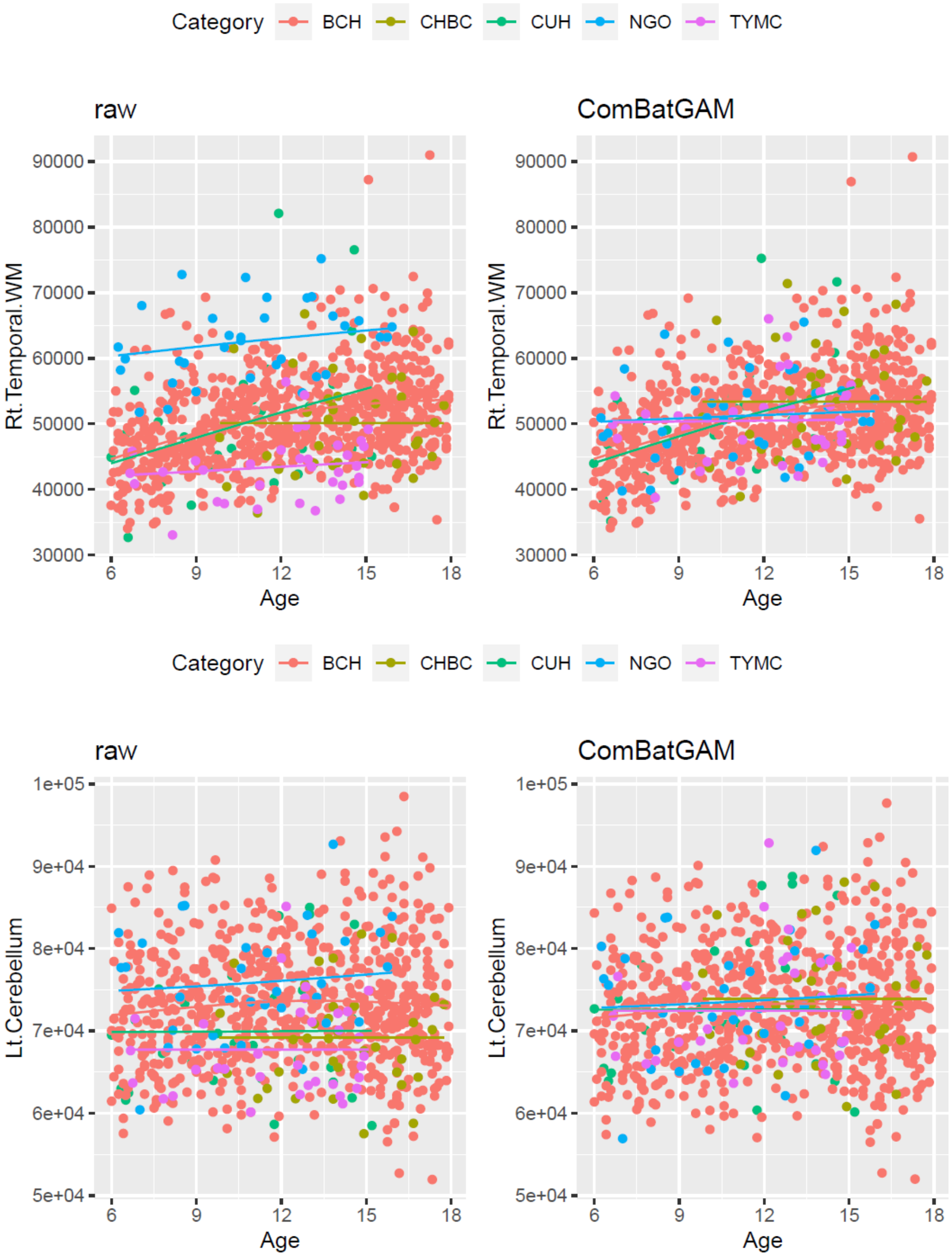
Category BCH CHBC CUH NGO TYMC
raw
ComBatGAM
Rt.Temporal.WM
90000
80000
70000
60000
50000
40000
30000
6
9
12
15
18
Age
Category BCH CHBC CUH NGO TYMC
raw
ComBatGAM
Lt.Cerebellum
1e+05
9e+04
8e+04
7e+04
6e+04
5e+04
Age

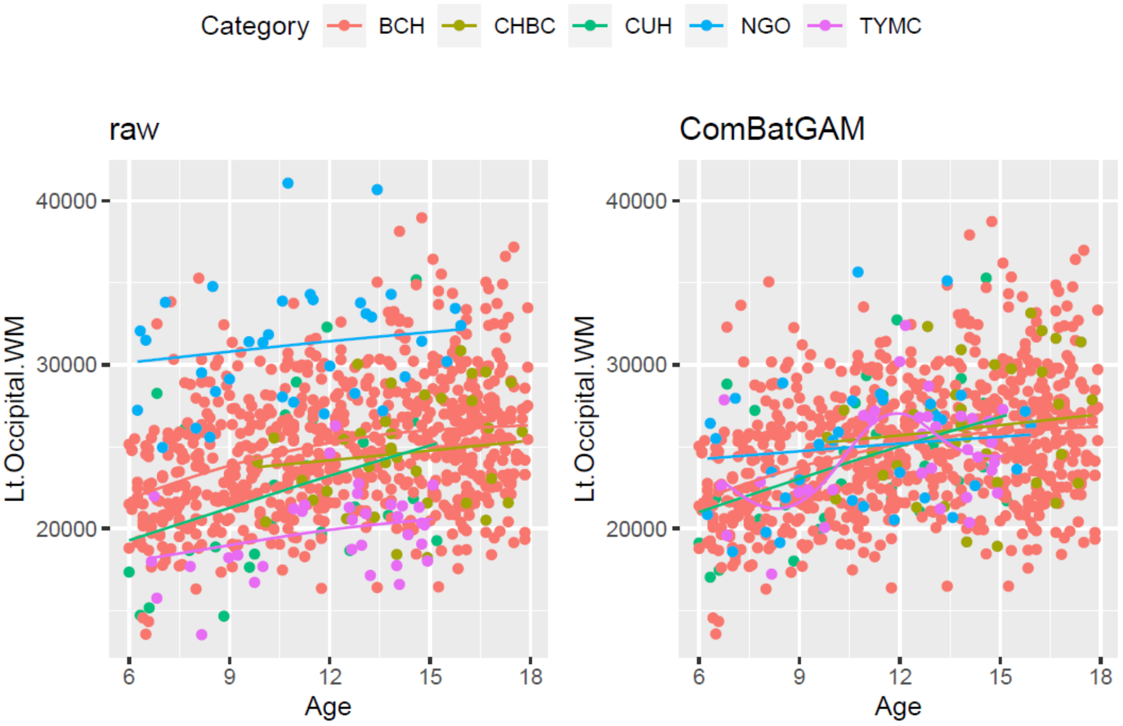
Category
BCH
CHBC
CUH
NGO
TYMC
raw
ComBatGAM
Lt.Occipital.WM
40000
30000
20000
6
9
12
15
18
Age

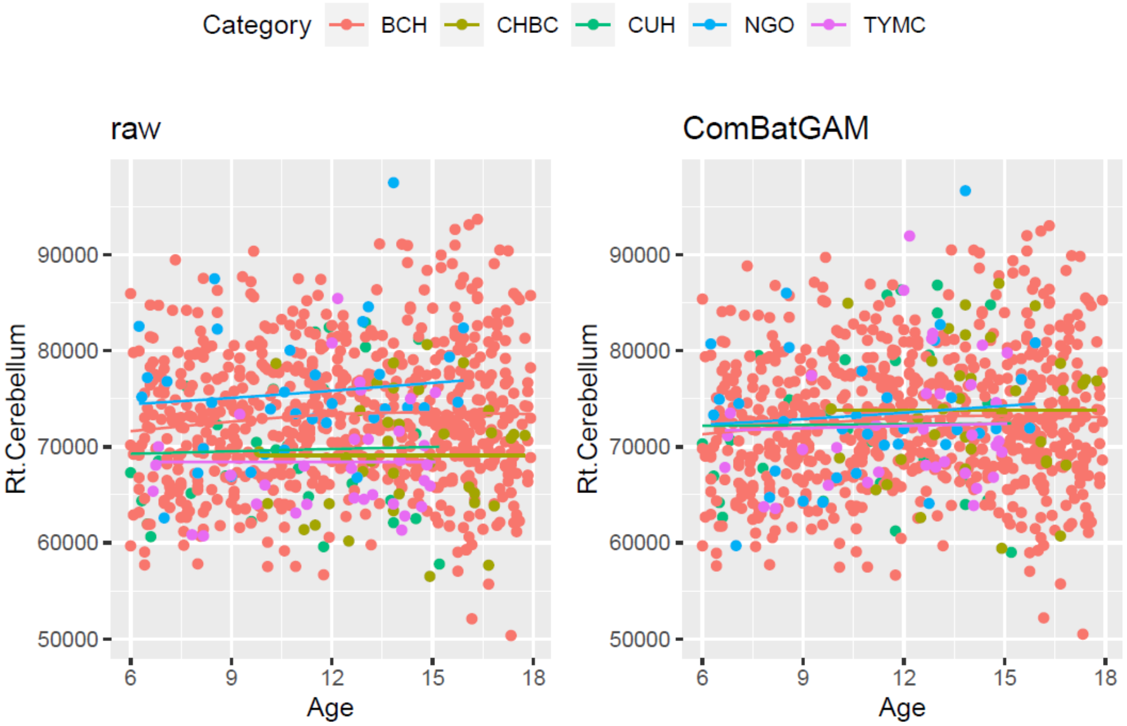
Category
BCH
CHBC
CUH
NGO
TYMC
raw
ComBatGAM
Rt.Cerebellum
90000
80000
70000
60000
50000
6
9
12
15
18
Age

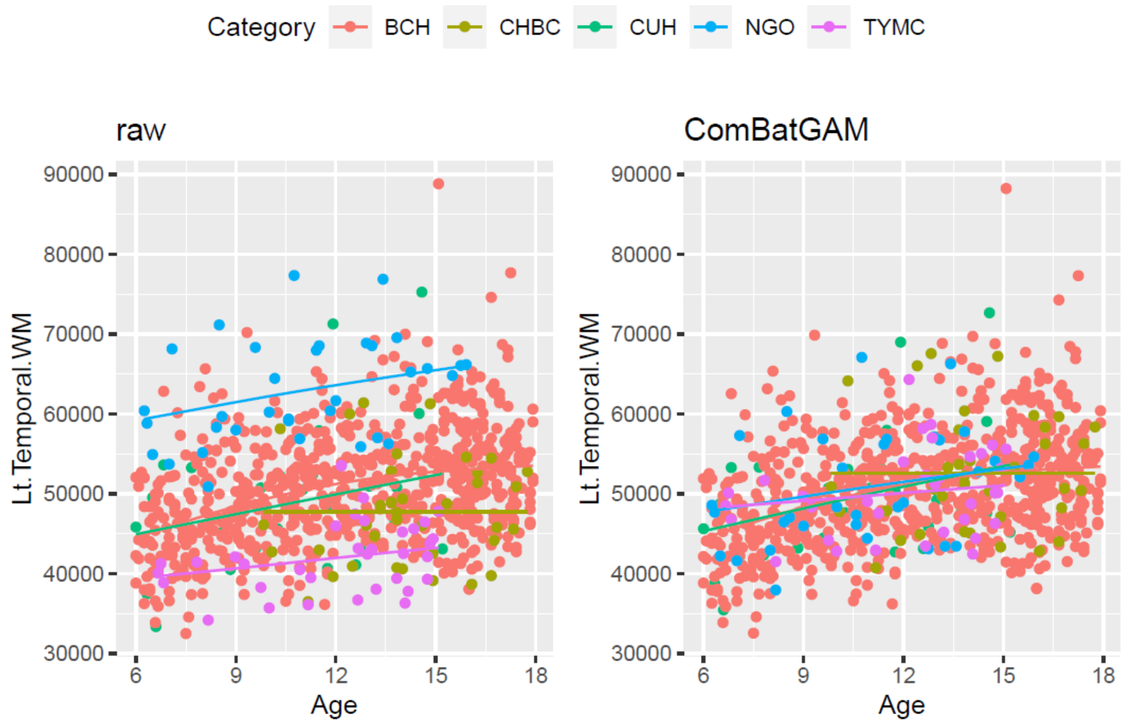
Category
BCH
CHBC
CUH
NGO
TYMC
raw
ComBatGAM
Lt.Temporal.WM
Age
90000
80000
70000
60000
50000
40000
30000
6
9
12
15
18

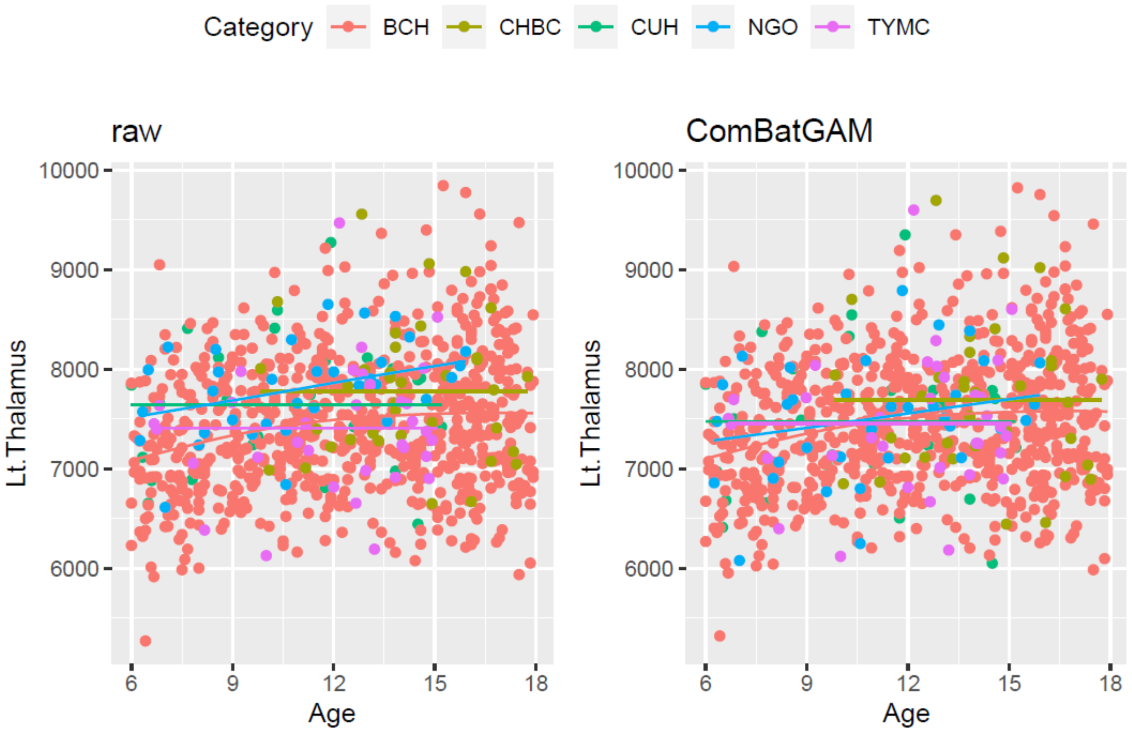
Category
BCH
CHBC
CUH
NGO
TYMC
raw
ComBatGAM
Lt.Thalamus
Age
10000
9000
8000
7000
6000
6
9
12
15
18

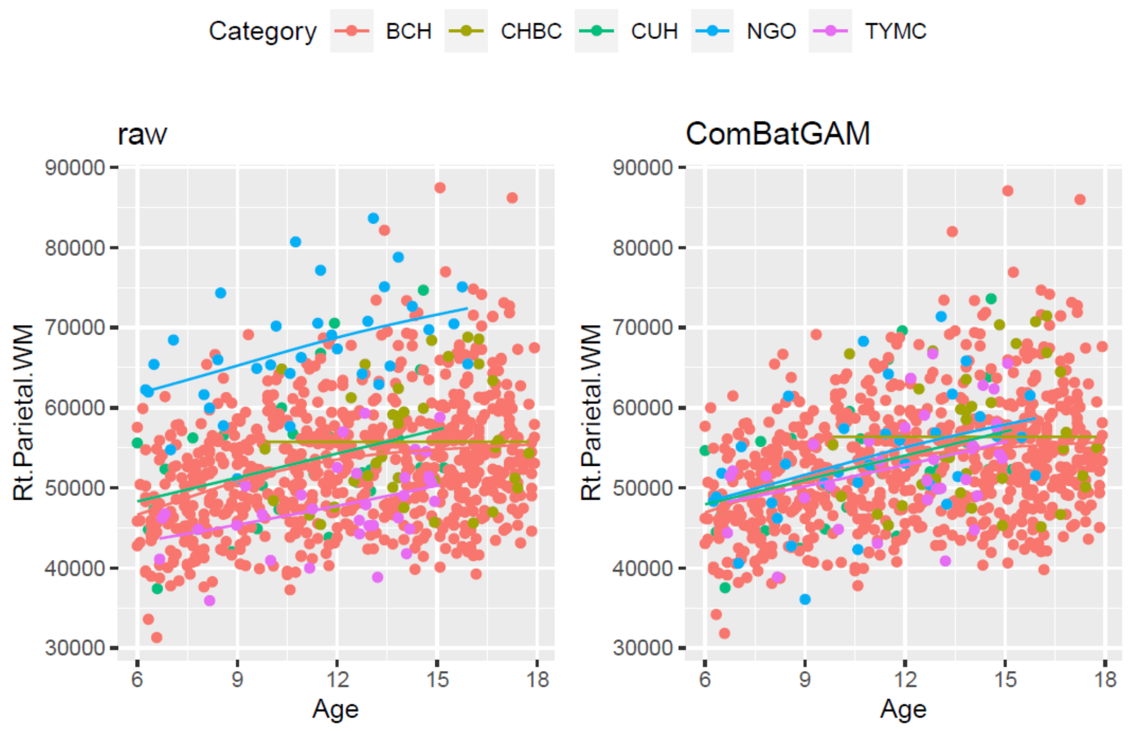
Category
BCH
CHBC
CUH
NGO
TYMC
raw
ComBatGAM
Rt.Parietal.WM
90000
80000
70000
60000
50000
40000
30000
6
9
12
15
18
Age

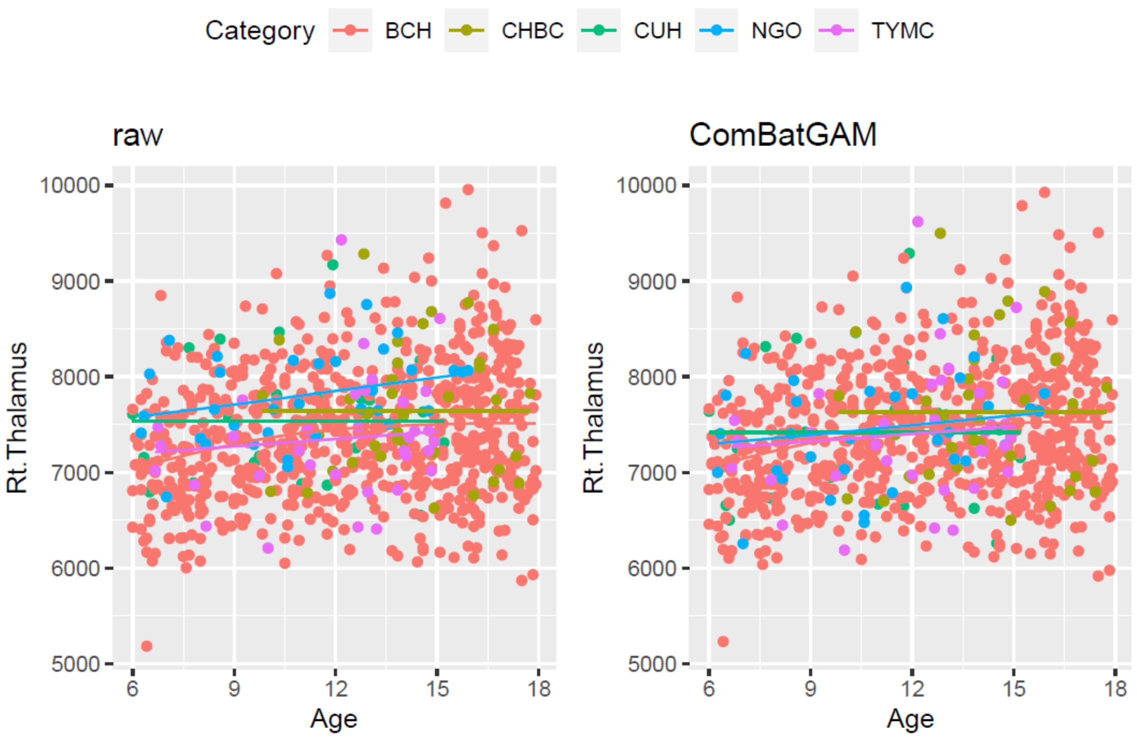
Category
BCH
CHBC
CUH
NGO
TYMC
raw
ComBatGAM
Rt.Thalamus
10000
9000
8000
7000
6000
5000
6
9
12
15
18
Age

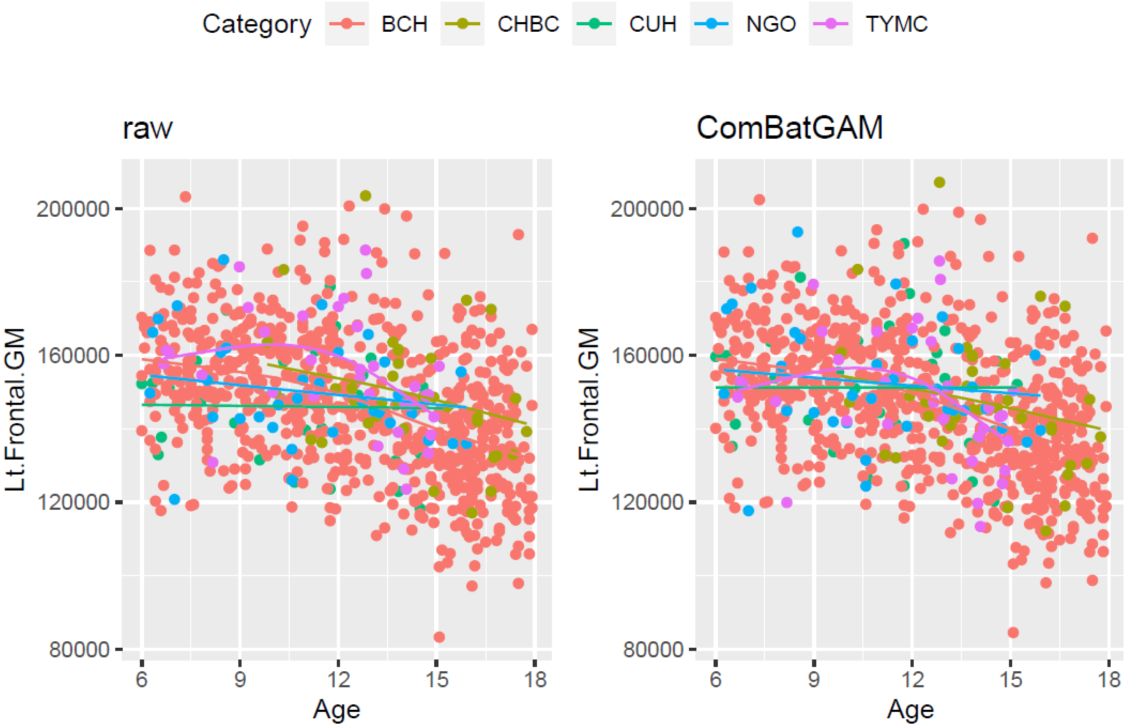
Category
BCH
CHBC
CUH
NGO
TYMC
raw
ComBatGAM
Lt.Frontal.GM
200000
160000
120000
80000
6
9
12
15
18
Age

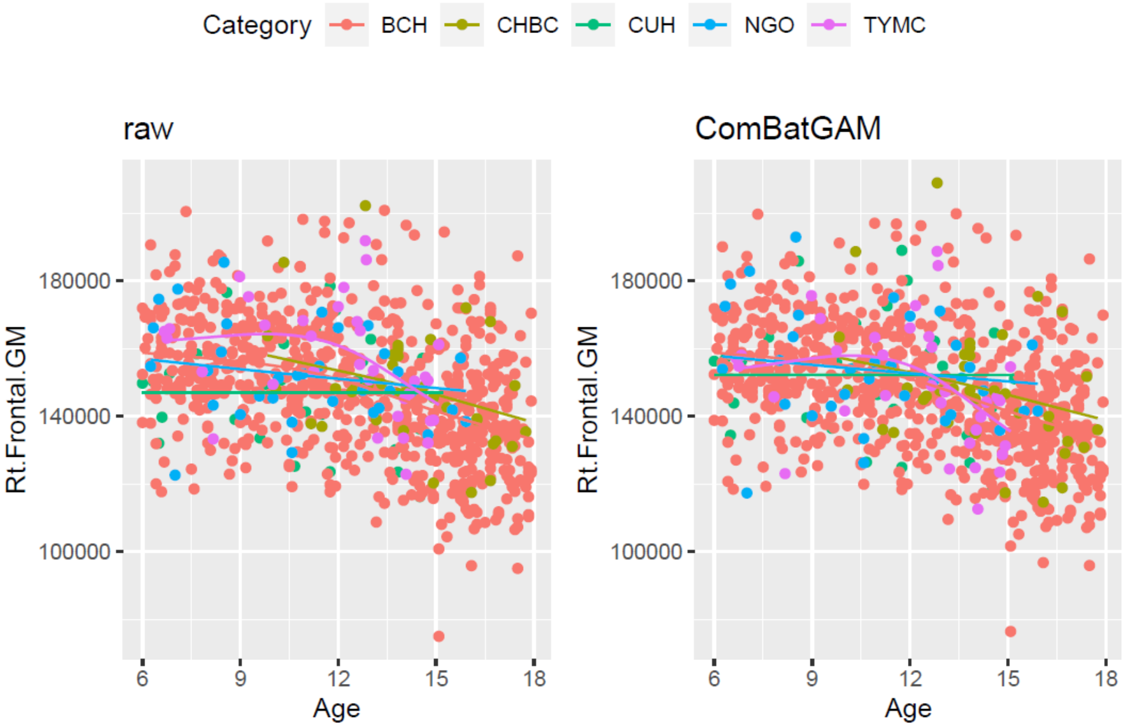
Category
BCH
CHBC
CUH
NGO
TYMC
raw
ComBatGAM
Rt.Frontal.GM
180000
140000
100000
6
9
12
15
18
Age

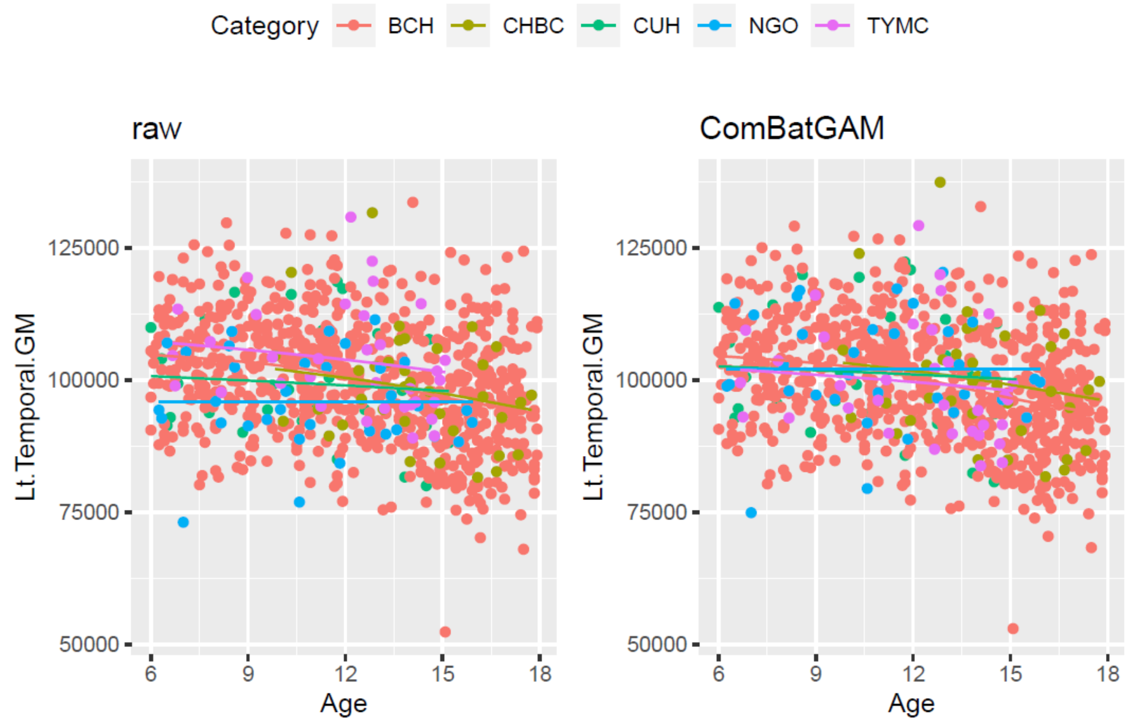
Category
BCH
CHBC
CUH
NGO
TYMC
raw
ComBatGAM
Lt.Temporal.GM
125000
100000
75000
50000
6
9
12
15
18
Age

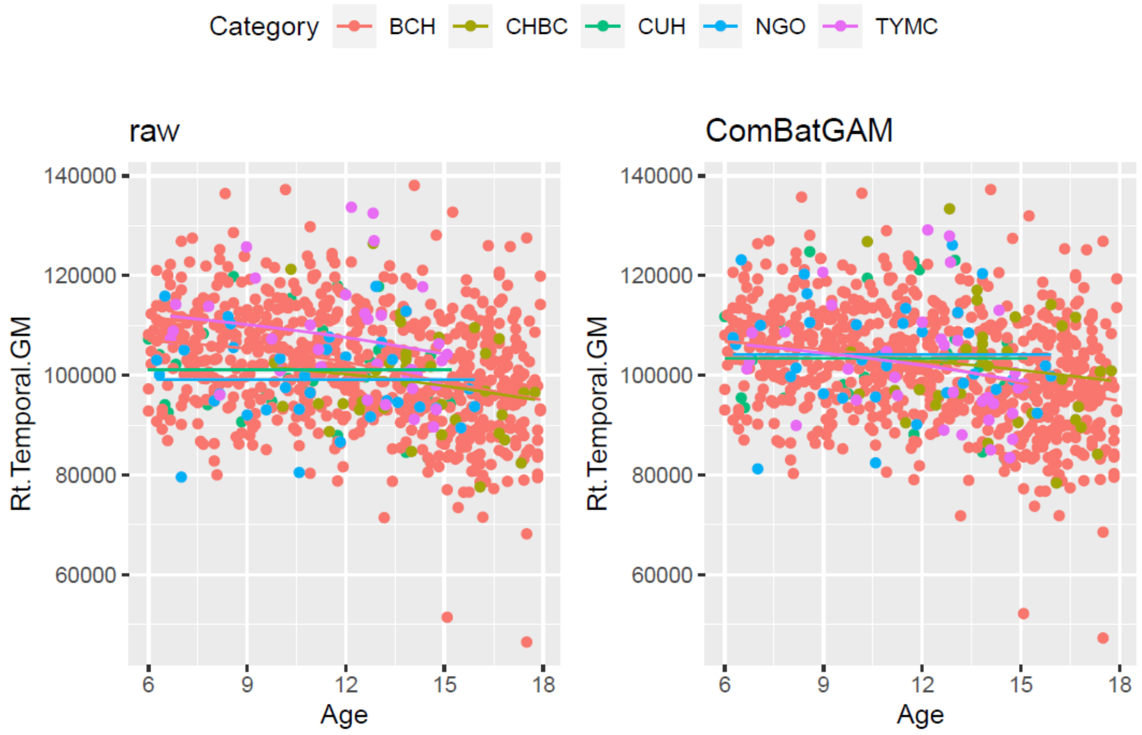
Category
BCH
CHBC
CUH
NGO
TYMC
raw
ComBatGAM
Rt.Temporal.GM
140000
120000
100000
80000
60000
6
9
12
15
18
Age

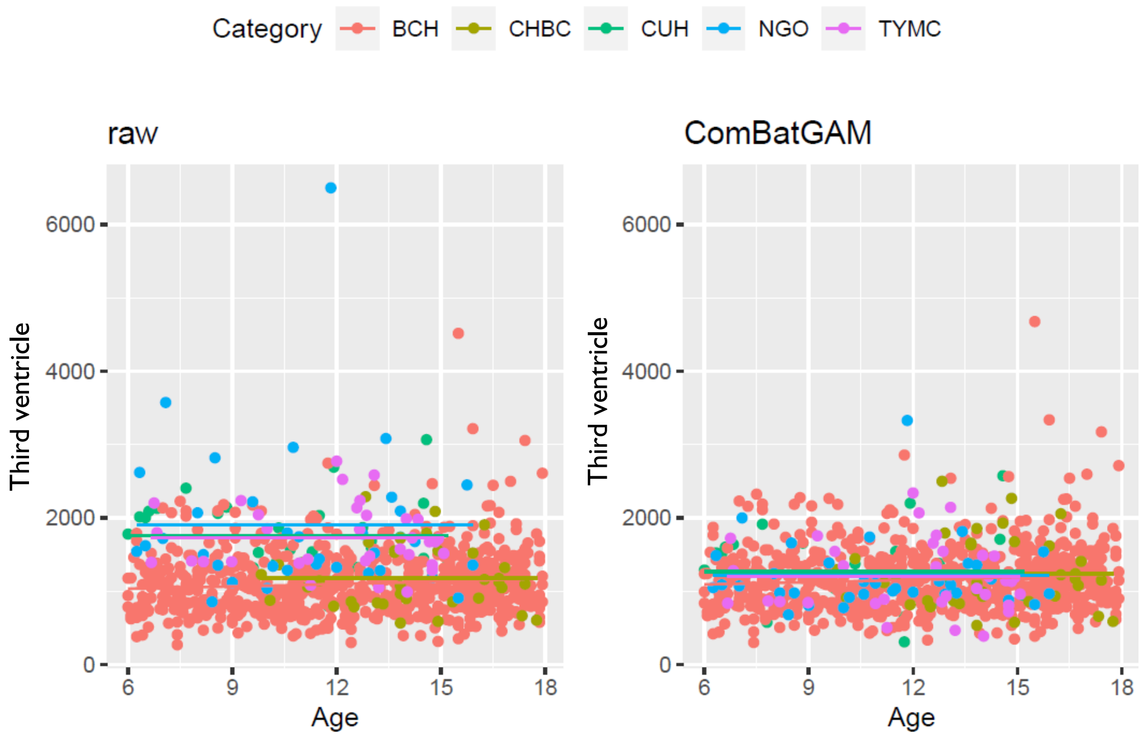
Category
BCH
CHBC
CUH
NGO
TYMC
raw
ComBatGAM
Third ventricle
0
2000
4000
6000
6
9
12
15
18
Age

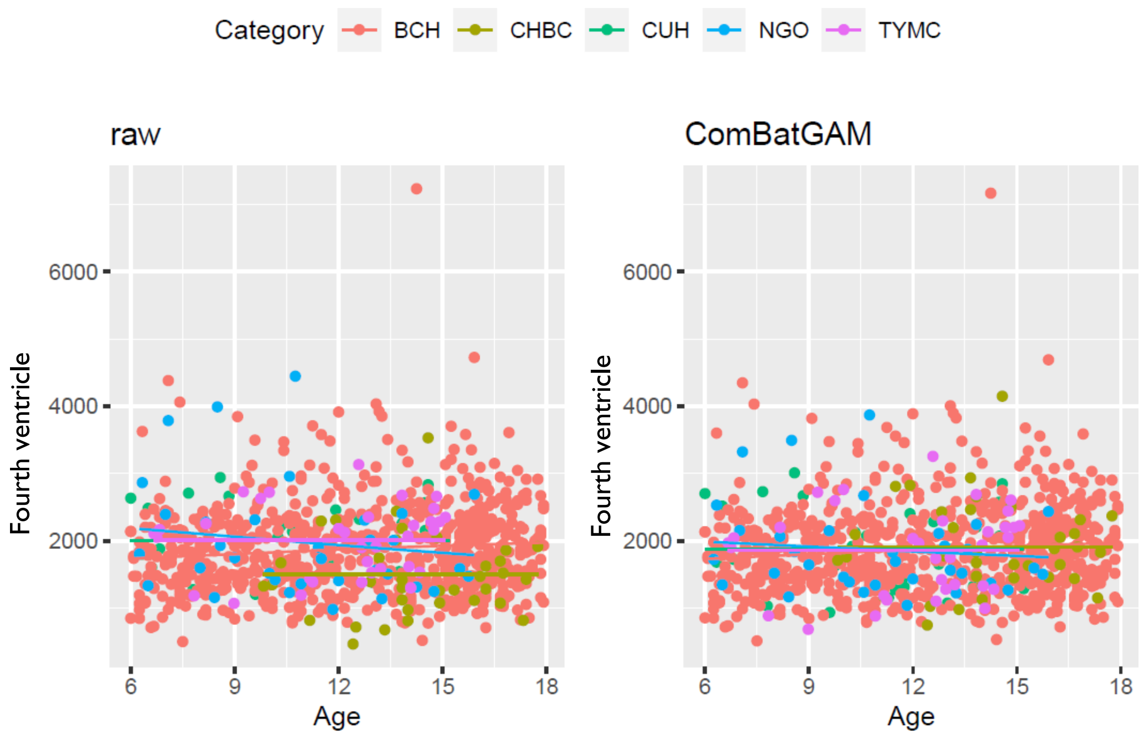
Category
BCH
CHBC
CUH
NGO
TYMC
raw
ComBatGAM
Fourth ventricle
2000
4000
6000
6
9
12
15
18
Age

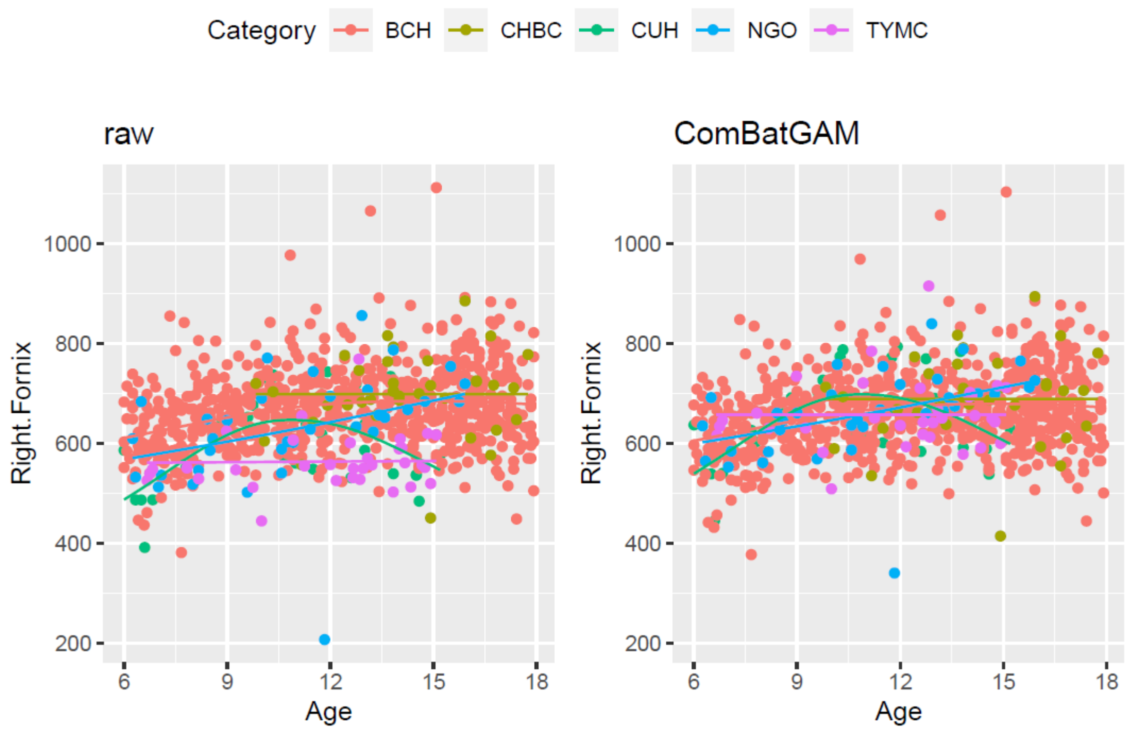
Category
BCH
CHBC
CUH
NGO
TYMC
raw
ComBatGAM
Right.Fornix
1000
800
600
400
200
6
9
12
15
18
Age

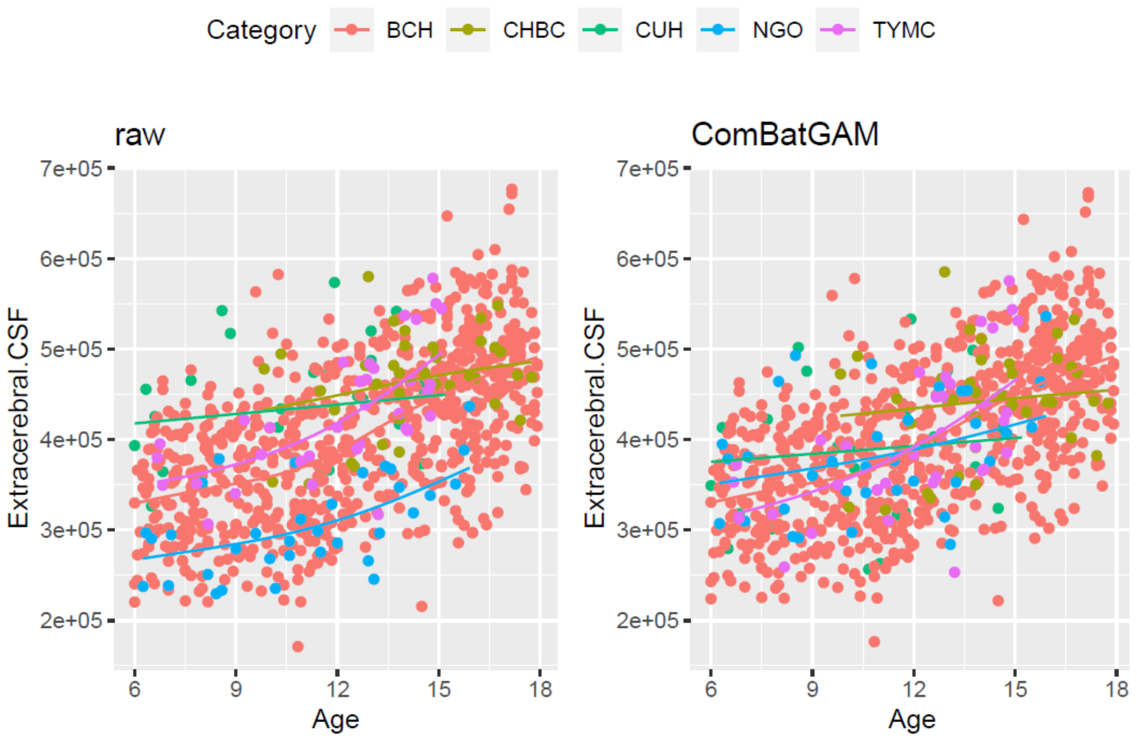
Category
BCH
CHBC
CUH
NGO
TYMC
raw
ComBatGAM
Extracerebral.CSF
7e+05
6e+05
5e+05
4e+05
3e+05
2e+05
6
9
12
15
18
Age
