# Supplementary material for: A Brain Morphometry Study with Across-Site Harmonization Using a ComBat-Generalized Additive Model in Children and Adolescents
Source: Diagnostics (Basel). 2023 Aug 27;13(17):2774. doi: 10.3390/diagnostics13172774 (PMC10487204; doi:10.3390/diagnostics13172774)

Figure S2: Regional volume of each part of the cortical gray and white matter

Scatter plots and regression lines (between age at scan and regional volume of cortical GM and WM) in male (blue circles and lines) and female (red circles and lines) neurotypical controls were shown. Abbreviations: GM, gray matter; Lt, left; Rt, right; WM, white matter.

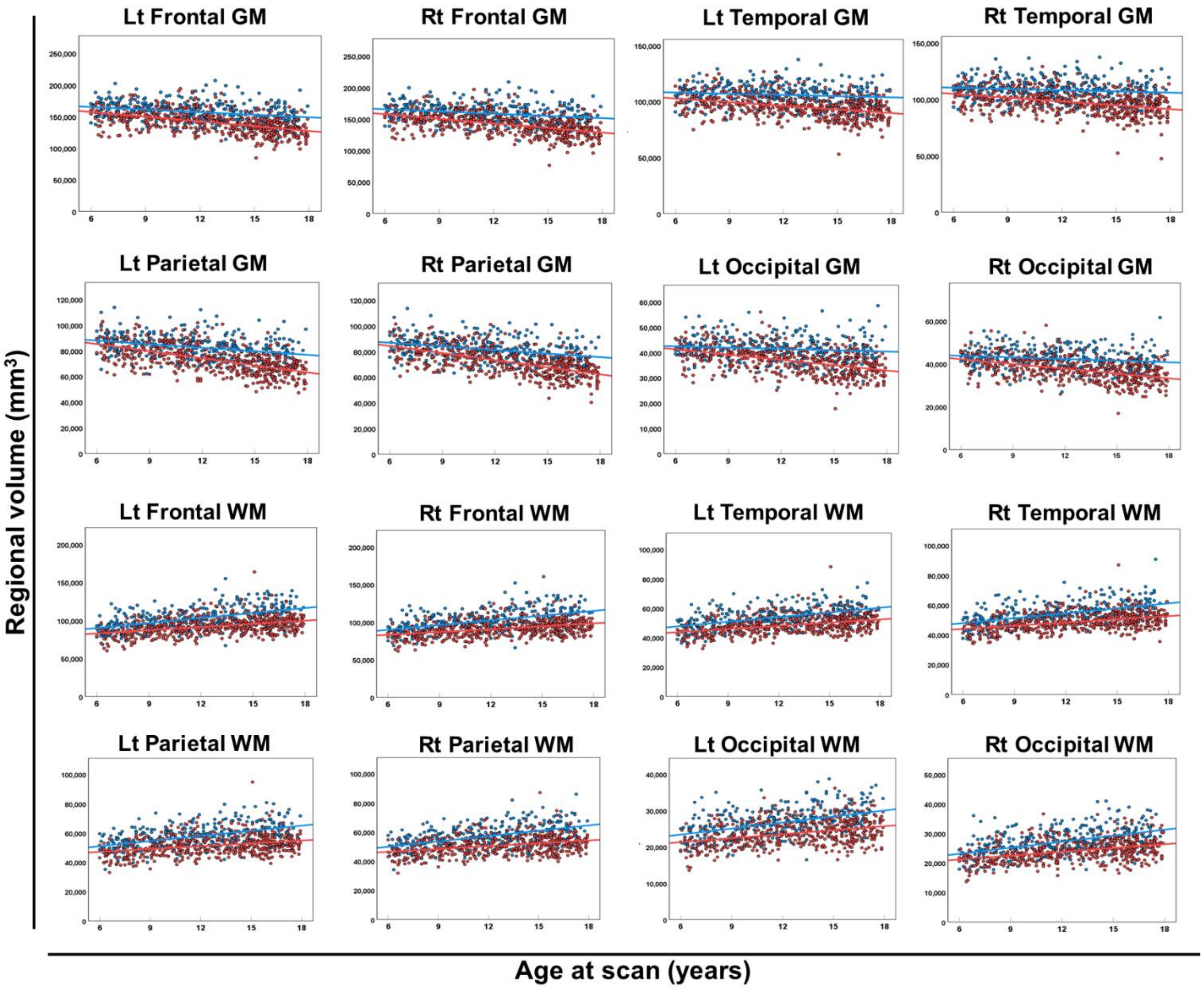

Supplement: Supplementary file 1 [file diagnostics-13-02774-s001.zip › BASH-NC Figure S2.pdf]
