# Supplementary material for: A Brain Morphometry Study with Across-Site Harmonization Using a ComBat-Generalized Additive Model in Children and Adolescents
Source: Diagnostics (Basel). 2023 Aug 27;13(17):2774. doi: 10.3390/diagnostics13172774 (PMC10487204; doi:10.3390/diagnostics13172774)

Figure S3: Regional volume of each subcortical gray matter part

Scatter plots and regression lines (between age at scan and regional volume of subcortical gray matter) in male (blue circles and lines) and female (red circles and lines) neurotypical controls are shown. Abbreviations: Lt, left; Rt, right.

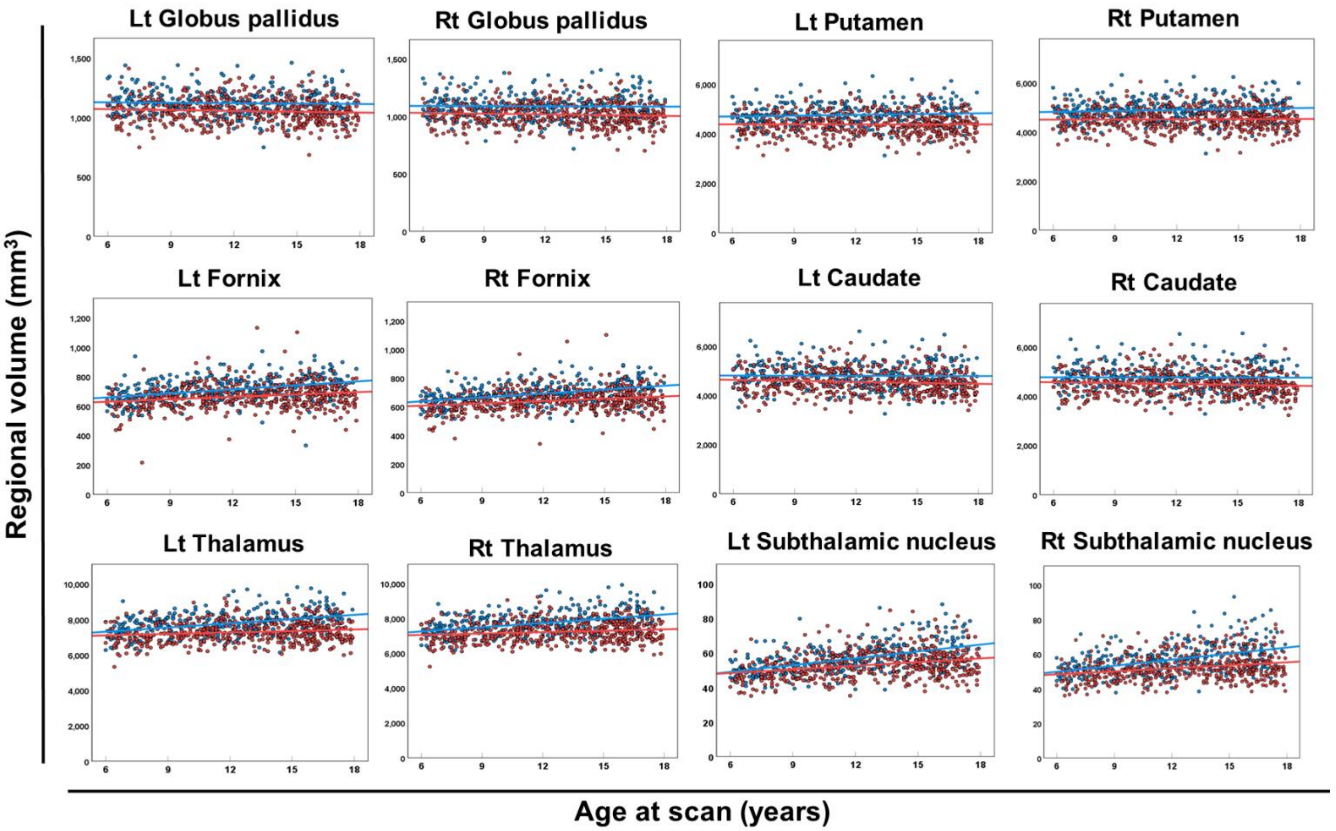

Supplement: Supplementary file 1 [file diagnostics-13-02774-s001.zip › BASH-NC Figure S3.pdf]
