# Supplementary material for: A Brain Morphometry Study with Across-Site Harmonization Using a ComBat-Generalized Additive Model in Children and Adolescents
Source: Diagnostics (Basel). 2023 Aug 27;13(17):2774. doi: 10.3390/diagnostics13172774 (PMC10487204; doi:10.3390/diagnostics13172774)

Figure S4: Regional volume of each part of cerebrospinal fluid system

Scatter plots and regression lines (between age at scan and regional volume of the cerebrospinal fluid system) in male (blue circles and lines) and female (red circles and lines) neurotypical controls were shown. Abbreviations: CSF, cerebrospinal fluid; Lt, left; Rt, right.

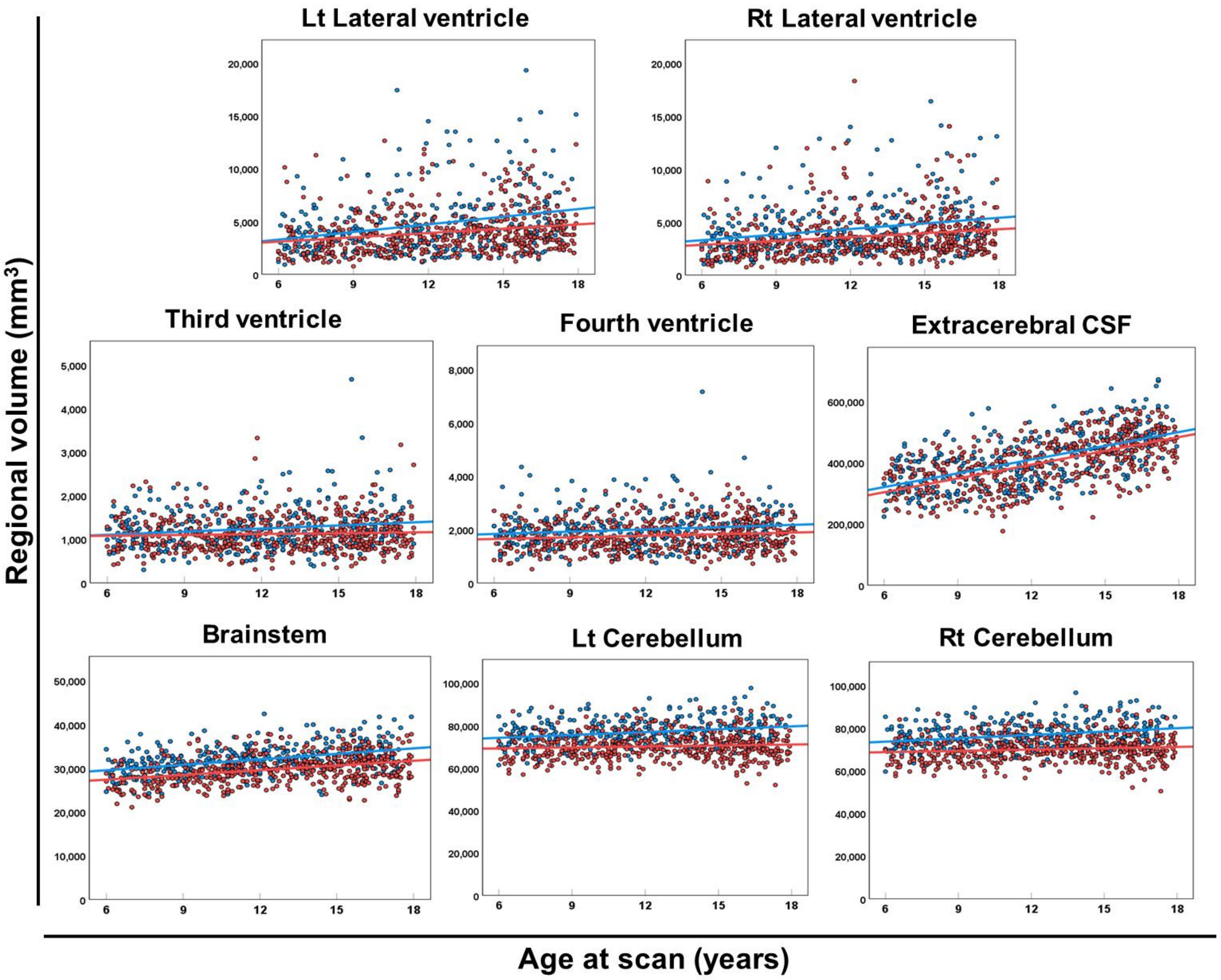

Supplement: Supplementary file 1 [file diagnostics-13-02774-s001.zip › BASH-NC Figure S4.pdf]
