# Supplementary material for: A Brain Morphometry Study with Across-Site Harmonization Using a ComBat-Generalized Additive Model in Children and Adolescents
Source: Diagnostics (Basel). 2023 Aug 27;13(17):2774. doi: 10.3390/diagnostics13172774 (PMC10487204; doi:10.3390/diagnostics13172774)
Supplement: Supplementary file 1 [file diagnostics-13-02774-s001.zip › BASH-NC Table S1.pdf]

Table S1. Brain volumetric measurements after harmonization for each age range.

		6YO<=Age<9YO							
		Male (N=89)		Female (N=86)		Total (N=175)		P value (male vs. female)	Absolute Cohen's d (male vs. female)
ANIMAL label	Description	Mean (mm^3)	SD (mm^3)	Mean (mm^3)	SD (mm^3)	Mean (mm^3)	SD (mm^3)		
Global	Whole brain*	1,819,754	142,320	1,682,695	143,133	1,752,399	158,031	$1.8x10^{-9}$	0.96
Global	Cortical GM*	801,934	63,252	745,662	71,998	774,280	73,155	$1.5x10^{-7}$	0.83
Global	WM*	436,705	52,331	390,500	42,780	413,998	53,063	$1.4x10^{-9}$	0.97
Global	SGM*	37,714	2,708	35,171	2,758	36,464	3,008	$5.2x10^{-9}$	0.93
Global	Extra-axial CSF	363,811	62,982	347,218	62,073	355,657	62,910	0.081	0.27
Global	CB*	179,590	14,080	164,145	13,832	172,000	15,928	$9.0x10^{-12}$	1.11
2	Rt Parietal GM*	85,555	8,633	80,327	9,216	82,986	9,276	$1.5x10^{-4}$	0.59
3	Lt Lateral ventricle	3,671	1,817	3,189	1,995	3,434	1,916	0.097	0.25
4	Rt Occipital GM*	43,665	4,797	40,710	5,344	42,213	5,271	$1.7x10^{-4}$	0.58
6	Lt Parietal GM*	86,685	8,836	80,968	9,540	83,876	9,600	$6.1x10^{-5}$	0.62
8	Lt Occipital GM*	42,559	4,379	39,814	4,812	41,210	4,786	$1.2x10^{-4}$	0.6
9	Rt Lateral ventricle	3,534	1,802	2,835	1,713	3,191	1,788	$9.3x10^{-3}$	0.4
11	Rt Globus pallidus*	1,103	101	1,012	101	1,058	110	$1.1x10^{-8}$	0.91
12	Lt Globus pallidus*	1,134	102	1,055	109	1,095	112	$1.7x10^{-6}$	0.75
14	Lt Putamen*	4,752	392	4,320	435	4,540	466	$1.0x10^{-10}$	1.04
16	Rt Putamen*	4,854	405	4,435	418	4,648	461	$2.4x10^{-10}$	1.02
17	Rt Frontal WM*	92,870	11,716	83,277	9,510	88,156	11,693	$1.5x10^{-8}$	0.9
20	Brainstem*	30,107	2,870	27,416	2,673	28,785	3,078	$1.3x10^{-9}$	0.97
23	Rt Subthalamic nucleus*	52	6	48	7	50	7	$1.1x10^{-4}$	0.6
29	Lt Fornix*	670	72	615	82	643	82	$5.2x10^{-6}$	0.71
30	Lt Frontal WM*	93,411	11,578	83,319	9,684	88,452	11,799	$3.0x10^{-9}$	0.94
33	Lt Subthalamic nucleus*	51	6	48	6	49	6	$1.6x10^{-4}$	0.58
39	Lt Caudate*	4,815	497	4,546	472	4,683	502	$3.3x10^{-4}$	0.55
45	Rt Occipital WM*	24,162	3,943	21,365	3,342	22,787	3,910	$1.0x10^{-6}$	0.76
53	Rt Caudate*	4,791	490	4,480	453	4,638	496	$2.2x10^{-5}$	0.66
57	Lt Parietal WM*	52,365	6,595	46,745	5,813	49,603	6,815	$1.2x10^{-8}$	0.9
59	Rt Temporal WM*	49,422	6,509	44,025	5,130	46,770	6,450	$7.1x10^{-9}$	0.92
67	Lt Cerebellum*	74,938	6,008	68,550	6,032	71,799	6,804	$4.9x10^{-11}$	1.06
73	Lt Occipital WM*	24,308	3,698	21,295	3,212	22,828	3,774	$3.8x10^{-8}$	0.87
76	Rt Cerebellum*	74,545	5,888	68,179	5,837	71,416	6,661	$2.0x10^{-11}$	1.09
83	Lt Temporal WM*	48,871	6,347	44,047	5,218	46,500	6,287	$1.4x10^{-7}$	0.83
102	Lt Thalamus*	7,441	563	7,037	612	7,243	620	$1.1x10^{-5}$	0.69
105	Rt Parietal WM*	51,296	6,294	46,426	5,413	48,903	6,349	$1.4x10^{-7}$	0.83
203	Rt Thalamus*	7,399	557	6,978	579	7,192	605	$2.2x10^{-6}$	0.74
210	Lt Frontal GM*	163,143	13,620	150,698	15,162	157,027	15,654	$5.1x10^{-8}$	0.86
211	Rt Frontal GM*	163,888	13,586	150,686	14,881	157,400	15,663	$6.1x10^{-9}$	0.93
218	Lt Temporal GM*	106,978	8,948	100,394	10,054	103,743	10,038	$9.3x10^{-6}$	0.69
219	Rt Temporal GM*	109,461	9,394	102,065	9,991	105,826	10,351	$1.2x10^{-6}$	0.76
232	Third ventricle	1,168	388	1,118	416	1,144	401	0.41	0.12
233	Fourth ventricle	1,877	660	1,652	518	1,767	603	0.013	0.38
254	Rt Fornix*	651	65	596	70	624	73	$1.8x10^{-7}$	0.82
255	Extracerebral CSF	353,561	61,724	338,424	60,408	346,122	61,376	0.10	0.25

* indicates a statistically significant finding based on $p < 1.4 \times 10^{-3}$ (two-tail unpaired t test). Abbreviations: CB, cerebellum and brainstem; GM, gray matter; CSF, extra-axial cerebrospinal fluid; SGM, Subcortical gray matter and fornix; WM, white matter.

Table S1. continued.

		9YO<=Age<12YO							
		Male (N=85)		Female (N=127)		Total (N=212)		P value (male vs. female)	Absolute Cohen's d (male vs. female)
ANIMAL label	Description	Mean (mm^3)	SD (mm^3)	Mean (mm^3)	SD (mm^3)	Mean (mm^3)	SD (mm^3)		
Global	Whole brain*	1,870,988	133,244	1,729,428	140,313	1,786,186	153,820	$4.0x10^{-12}$	1.03
Global	Cortical GM*	787,813	63,495	733,343	70,839	755,183	72,922	$2.2x10^{-8}$	0.8
Global	WM*	473,186	44,321	424,176	47,318	443,826	51,949	$8.5x10^{-13}$	1.06
Global	SGM*	38,328	2,530	36,241	3,024	37,078	3,010	$1.6x10^{-7}$	0.74
Global	Extra-axial CSF	388,022	73,526	365,506	69,591	374,534	71,878	0.027	0.32
Global	CB*	183,639	13,842	170,163	14,034	175,566	15,418	$7.9x10^{-11}$	0.97
2	Rt Parietal GM*	82,873	8,966	77,527	8,638	79,670	9,135	$2.6x10^{-5}$	0.61
3	Lt Lateral ventricle	4,339	2,699	3,930	2,240	4,094	2,436	0.25	0.17
4	Rt Occipital GM*	42,524	4,740	39,344	5,140	40,619	5,212	$6.8x10^{-6}$	0.64
6	Lt Parietal GM*	84,153	8,966	78,671	9,204	80,869	9,479	$2.6x10^{-5}$	0.6
8	Lt Occipital GM*	41,459	4,797	38,692	4,758	39,801	4,953	$5.6x10^{-5}$	0.58
9	Rt Lateral ventricle	4,143	2,311	3,693	2,343	3,873	2,335	0.17	0.19
11	Rt Globus pallidus*	1,082	104	1,023	96	1,047	103	$5.5x10^{-5}$	0.59
12	Lt Globus pallidus*	1,124	100	1,061	109	1,086	110	$2.3x10^{-5}$	0.6
14	Lt Putamen*	4,757	413	4,409	470	4,548	478	$4.3x10^{-8}$	0.78
16	Rt Putamen*	4,876	427	4,520	460	4,663	479	$3.3x10^{-8}$	0.8
17	Rt Frontal WM*	99,957	9,329	89,961	10,426	93,968	11,121	$7.7x10^{-12}$	1
20	Brainstem*	31,568	2,810	29,283	2,884	30,199	3,061	$3.9x10^{-8}$	0.8
23	Rt Subthalamic nucleus*	55	6	51	7	53	7	$5.6x10^{-4}$	0.48
29	Lt Fornix*	712	72	671	79	687	79	$1.2x10^{-4}$	0.54
30	Lt Frontal WM*	100,644	9,867	90,248	10,374	94,416	11,363	$5.6x10^{-12}$	1.02
33	Lt Subthalamic nucleus	54	7	52	7	53	7	0.023	0.31
39	Lt Caudate	4,848	424	4,633	538	4,719	505	$1.3x10^{-3}$	0.44
45	Rt Occipital WM*	26,370	3,663	23,567	3,451	24,691	3,788	$8.8x10^{-8}$	0.79
53	Rt Caudate	4,783	438	4,585	529	4,664	503	$3.3x10^{-3}$	0.4
57	Lt Parietal WM*	57,159	6,644	51,299	6,716	53,649	7,266	$2.6x10^{-9}$	0.88
59	Rt Temporal WM*	53,560	5,676	47,862	5,482	50,147	6,213	$1.2x10^{-11}$	1.02
67	Lt Cerebellum*	76,104	5,952	70,584	6,066	72,797	6,590	$5.2x10^{-10}$	0.92
73	Lt Occipital WM*	25,892	3,248	23,438	3,204	24,422	3,433	$1.9x10^{-7}$	0.76
76	Rt Cerebellum*	75,968	6,020	70,296	6,002	72,570	6,611	$2.2x10^{-10}$	0.94
83	Lt Temporal WM*	53,369	5,455	47,429	5,364	49,811	6,127	$4.4x10^{-13}$	1.1
102	Lt Thalamus*	7,703	570	7,310	580	7,468	606	$2.3x10^{-6}$	0.68
105	Rt Parietal WM*	56,236	6,010	50,372	6,482	52,723	6,912	$1.8x10^{-10}$	0.93
203	Rt Thalamus*	7,647	581	7,281	601	7,428	618	$1.6x10^{-5}$	0.62
210	Lt Frontal GM*	159,431	13,719	149,075	15,366	153,227	15,550	$7.0x10^{-7}$	0.7
211	Rt Frontal GM*	160,558	13,747	150,187	15,272	154,345	15,507	$6.5x10^{-7}$	0.71
218	Lt Temporal GM*	107,533	8,762	98,992	9,499	102,417	10,102	$2.0x10^{-10}$	0.93
219	Rt Temporal GM*	109,282	9,064	100,856	9,354	104,235	10,104	$5.6X10^{-10}$	0.91
232	Third ventricle	1,168	426	1,102	440	1,128	435	0.27	0.15
233	Fourth ventricle	1,964	689	1,729	592	1,823	641	0.011	0.37
254	Rt Fornix*	687	65	645	76	662	74	$2.8x10^{-5}$	0.58
255	Extracerebral CSF	376,408	71,976	355,054	68,372	363,615	70,456	0.032	0.31

*indicates a statistically significant finding based on $p < 1.4 \times 10^{-3}$ (two-tail unpaired t test). Abbreviations: CB, cerebellum and brainstem; GM, gray matter; CSF, extra-axial cerebrospinal fluid; SGM, Subcortical gray matter and fornix; WM, white matter.

Table S1. continued.

		12YO<=Age<15YO							
		Male (N=93)		Female (N=115)		Total (N=208)		P value (male vs. female)	Absolute Cohen's d (male vs. female)
ANIMAL label	Description	Mean (mm^3)	SD (mm^3)	Mean (mm^3)	SD (mm^3)	Mean (mm^3)	SD (mm^3)		
Global	Whole brain*	1,921,579	164,698	1,768,993	116,849	1,837,216	159,244	$3.4x10^{-12}$	1.09
Global	Cortical GM*	767,612	76,019	681,028	61,996	719,741	80,915	$8.6x10^{-16}$	1.26
Global	WM*	493,138	60,699	443,730	43,184	465,821	57,192	$5.3x10^{-10}$	0.95
Global	SGM*	38,053	3,318	36,274	2,567	37,070	3,051	$3.6x10^{-5}$	0.61
Global	Extra-axial CSF	435,120	78,133	435,147	66,097	435,135	71,548	0.998	$3.8x10^{-4}$
Global	CB*	187,656	14,739	172,814	12,441	179,450	15,379	$7.1x10^{-13}$	1.01
2	Rt Parietal GM*	79,328	8,904	69,982	8,005	74,161	9,604	$2.8x10^{-13}$	1.11
3	Lt Lateral ventricle	4,924	3,099	3,841	1,756	4,326	2,501	$3.2x10^{-3}$	0.44
4	Rt Occipital GM*	42,238	4,710	36,501	4,415	39,066	5,364	$2.5x10^{-16}$	1.26
6	Lt Parietal GM*	80,533	9,043	71,239	8,415	75,395	9,839	$1.3x10^{-12}$	1.07
8	Lt Occipital GM*	41,198	4,832	35,653	4,185	38,132	5,259	$1.5x10^{-15}$	1.24
9	Rt Lateral ventricle	4,425	2,464	3,528	2,023	3,929	2,270	$5.3x10^{-3}$	0.4
11	Rt Globus pallidus	1,065	112	1,031	103	1,046	108	0.024	0.32
12	Lt Globus pallidus	1,093	114	1,071	106	1,081	110	0.15	0.21
14	Lt Putamen*	4,707	519	4,441	441	4,560	494	$1.2x10^{-4}$	0.56
16	Rt Putamen*	4,831	515	4,584	452	4,694	495	$3.5x10^{-4}$	0.52
17	Rt Frontal WM*	104,437	14,795	94,534	10,656	98,962	13,571	$2.2x10^{-7}$	0.78
20	Brainstem*	32,504	3,175	30,589	2,786	31,445	3,109	$9.1x10^{-6}$	0.65
23	Rt Subthalamic nucleus*	58	8	54	8	56	8	$3.8x10^{-4}$	0.51
29	Lt Fornix	714	77	682	84	696	82	$5.9x10^{-3}$	0.38
30	Lt Frontal WM*	105,080	14,737	95,325	10,439	99,687	13,423	$2.5x10^{-7}$	0.78
33	Lt Subthalamic nucleus	59	8	56	8	57	8	0.009	0.37
39	Lt Caudate	4,680	557	4,564	431	4,616	494	0.1	0.24
45	Rt Occipital WM*	28,066	4,309	24,920	3,009	26,326	3,962	$1.6x10^{-8}$	0.86
53	Rt Caudate	4,649	547	4,492	440	4,562	496	0.026	0.32
57	Lt Parietal WM*	58,993	7,668	52,628	5,860	55,474	7,423	$5.2x10^{-10}$	0.95
59	Rt Temporal WM*	55,369	6,650	50,186	5,340	52,503	6,483	$6.9x10^{-9}$	0.87
67	Lt Cerebellum*	77,551	6,315	71,095	5,403	73,982	6,645	$4.3x10^{-13}$	1.11
73	Lt Occipital WM*	27,551	3,957	24,433	2,846	25,827	3,719	$1.8x10^{-9}$	0.92
76	Rt Cerebellum*	77,601	6,394	71,131	5,274	74,024	6,625	$4.0x10^{-13}$	1.12
83	Lt Temporal WM*	55,097	6,535	49,855	5,372	52,199	6,457	$3.5x10^{-9}$	0.89
102	Lt Thalamus*	7,778	691	7,350	555	7,541	654	$2.8x10^{-6}$	0.69
105	Rt Parietal WM*	58,546	7,731	51,849	5,635	54,843	7,429	$6.8x10^{-11}$	1.01
203	Rt Thalamus*	7,723	676	7,290	550	7,484	645	$1.4x10^{-6}$	0.71
210	Lt Frontal GM*	154,922	17,945	138,376	14,241	145,774	17,967	$1.4x10^{-11}$	1.03
211	Rt Frontal GM*	156,430	17,935	139,250	14,622	146,932	18,277	$4.0x10^{-12}$	1.06
218	Lt Temporal GM*	105,442	9,977	93,970	8,199	99,099	10,675	$5.7x10^{-16}$	1.27
219	Rt Temporal GM*	107,521	10,333	96,058	8,338	101,183	10,880	$3.0x10^{-15}$	1.24
232	Third ventricle	1,267	501	1,101	356	1,175	434	$8.1x10^{-3}$	0.39
233	Fourth ventricle	2,057	911	1,826	592	1,929	758	0.036	0.31
254	Rt Fornix*	695	76	659	70	675	75	$5.1x10^{-4}$	0.5
255	Extracerebral CSF	422,447	77,063	424,851	65,758	423,776	70,868	0.81	0.03

* indicates a statistically significant finding based on $p < 1.4 x 10^{-3}$ (two-tail unpaired t test). Abbreviations: CB, cerebellum and brainstem; GM, gray matter; CSF, extra-axial cerebrospinal fluid; SGM, Subcortical gray matter and fornix; WM, white matter.

Table S1. continued.

		15YO<=Age<18YO							
		Male (N=72)		Female (N=179)		Total (N=251)		P value (male vs. female)	Absolute Cohen's d (male vs. female)
ANIMAL label	Description	Mean (mm^3)	SD (mm^3)	Mean (mm^3)	SD (mm^3)	Mean (mm^3)	SD (mm^3)		
Global	Whole brain*	2,014,618	148,885	1,775,840	133,929	1,844,334	175,433	7.8x10^{-22}	1.73
Global	Cortical GM*	751,846	70,895	645,060	67,774	675,692	83,905	5.8x10^{-20}	1.55
Global	WM*	528,250	53,938	449,966	50,174	472,422	62,268	4.6x10^{-19}	1.53
Global	SGM*	39,862	3,081	35,853	2,632	37,003	3,306	1.2x10^{-16}	1.45
Global	Extra-axial CSF	502,777	80,760	473,363	62,903	481,801	69,622	6.6x10^{-3}	0.43
Global	CB*	191,883	15,048	171,598	15,155	177,417	17,674	5.7x10^{-17}	1.34
2	Rt Parietal GM*	77,541	8,496	64,905	8,029	68,530	9,960	1.2x10^{-19}	1.55
3	Lt Lateral ventricle	5,974	3,642	4,595	2,253	4,991	2,788	3.6x10^{-3}	0.51
4	Rt Occipital GM*	40,855	5,423	34,424	4,779	36,269	5,754	1.5x10^{-14}	1.29
6	Lt Parietal GM*	78,734	9,326	65,734	8,153	69,463	10,331	3.3x10^{-18}	1.53
8	Lt Occipital GM*	40,513	5,543	34,053	4,631	35,906	5,707	2.5x10^{-14}	1.32
9	Rt Lateral ventricle	5,305	3,229	4,193	2,222	4,512	2,594	8.7x10^{-3}	0.44
11	Rt Globus pallidus*	1,104	110	1,000	98	1,030	112	1.6x10^{-10}	1.03
12	Lt Globus pallidus*	1,135	94	1,037	101	1,065	109	1.3x10^{-11}	1
14	Lt Putamen*	4,863	481	4,357	424	4,502	496	2.8x10^{-12}	1.15
16	Rt Putamen*	4,999	472	4,488	432	4,635	500	1.2x10^{-12}	1.15
17	Rt Frontal WM*	112,013	12,794	95,341	10,754	100,123	13,635	1.1x10^{-16}	1.47
20	Brainstem*	33,847	3,551	30,866	3,248	31,721	3,594	9.7x10^{-9}	0.89
23	Rt Subthalamic nucleus*	62	10	54	8	56	9	8.6x10^{-10}	1.02
29	Lt Fornix*	758	92	678	80	701	91	3.1x10^{-9}	0.95
30	Lt Frontal WM*	113,619	13,091	96,631	11,267	101,504	14,083	1.5x10^{-16}	1.44
33	Lt Subthalamic nucleus*	63	10	55	8	57	9	4.1x10^{-9}	0.96
39	Lt Caudate*	4,887	573	4,469	458	4,589	528	2.2x10^{-7}	0.85
45	Rt Occipital WM*	30,131	3,932	25,397	3,677	26,755	4,315	1.1x10^{-14}	1.26
53	Rt Caudate*	4,868	564	4,428	444	4,554	520	3.9x10^{-8}	0.91
57	Lt Parietal WM*	63,317	7,245	53,253	6,650	56,140	8,197	5.4x10^{-18}	1.47
59	Rt Temporal WM*	59,346	6,776	50,978	6,140	53,378	7,367	2.5x10^{-15}	1.32
67	Lt Cerebellum*	78,905	6,708	70,330	6,357	72,790	7,527	6.0x10^{-16}	1.33
73	Lt Occipital WM*	28,898	3,564	24,872	3,471	26,027	3,939	2.8x10^{-13}	1.15
76	Rt Cerebellum*	79,131	6,456	70,402	6,494	72,906	7,584	4.7x10^{-17}	1.35
83	Lt Temporal WM*	58,330	5,649	50,804	6,024	52,963	6,821	1.8x10^{-16}	1.27
102	Lt Thalamus*	8,202	635	7,346	579	7591	710	2.8x10^{-17}	1.44
105	Rt Parietal WM*	62,597	7,152	52,691	6,328	55,532	7,950	5.1x10^{-18}	1.51
203	Rt Thalamus*	8,185	641	7,285	577	7,543	722	2.7x10^{-18}	1.51
210	Lt Frontal GM*	151,139	15,775	130,398	14,165	136,347	17,375	9.5x10^{-17}	1.42
211	Rt Frontal GM*	153,527	15,901	131,468	14,607	137,796	17,991	5.8x10^{-18}	1.47
218	Lt Temporal GM*	103,703	9,552	91,316	9,382	94,869	10,959	3.7x10^{-16}	1.31
219	Rt Temporal GM*	105,835	9,738	92,762	10,205	96,512	11,670	1.0x10^{-16}	1.3
232	Third ventricle	1,389	616	1,166	398	1,230	480	5.3x10^{-3}	0.48
233	Fourth ventricle	2,127	656	1,866	578	1,940	612	3.9x10^{-3}	0.43
254	Rt Fornix*	736	77	657	77	680	84	1.9x10^{-11}	1.02
255	Extracerebral CSF	487,983	80,712	461,544	61,565	469,128	68,500	0.014	0.39

* indicates a statistically significant finding based on $p < 1.4 \times 10^{-3}$ (two-tail unpaired t test). Abbreviations: CB, cerebellum and brainstem; GM, gray matter; CSF, extra-axial cerebrospinal fluid; SGM, Subcortical gray matter and fornix; WM, white matter.
