# Supplementary material for: A Brain Morphometry Study with Across-Site Harmonization Using a ComBat-Generalized Additive Model in Children and Adolescents
Source: Diagnostics (Basel). 2023 Aug 27;13(17):2774. doi: 10.3390/diagnostics13172774 (PMC10487204; doi:10.3390/diagnostics13172774)
Supplement: Supplementary file 1 [file diagnostics-13-02774-s001.zip › BASH-NC Table S2.pdf]

Table S2: Z-standardized scores of volumetric measurements of each additional case

	Case	1	2	3	4	5	6	7	8	9	10	11	12	13	14	15	16	17	18
	Sex	M	M	M	M	M	M	M	M	F	F	F	F	F	F	F	F	F	F
	Age at scan (years)	9.6	13.3	13.7	10.3	9.1	9.4	8.6	14	8.8	15.2	13.8	6.7	6.6	7.3	11.1	9.6	15.4	8.3
	Disorder	GS	GS	GS	MS	PHTS	PHTS	PHTS	PHTS	NC	NC	NC	RTT	RTT	RTT	RTT	RTT	RTT	RTT
	Scan-site	CUH	CUH	CUH	CUH	BCH	BCH	BCH	BCH	CHBC	CHBC	CHBC	BCH	BCH	BCH	BCH	CHBC	CHBC	CHBC
**ANIMAL label**	**Description**																		
Global	Whole brain	7.0	2.5	2.9	6.7	10.2	8.9	6.2	5.7	-0.7	0.4	-0.1	-1.2	0.6	-0.6	-2.8	-3.7	-4.4	-2.8
Global	Cortical GM	10.7	2.7	4.6	6.0	9.3	5.4	5.9	4.4	-0.1	1.5	1.1	-1.3	1.9	0.2	-4.7	-4.5	-4.1	-4.3
Global	White matter	1.6	0.7	1.5	5.6	13.8	10.1	4.9	5.1	0.3	0.4	-1.0	-2.0	-1.2	-0.3	-2.2	-2.1	-2.5	-1.3
Global	Subcortical GM	4.8	2.1	3.0	7.5	9.8	7.2	7.5	3.5	-0.3	-0.7	-0.6	-0.8	0.4	-0.2	-3.6	-2.6	-3.9	-2.9
Global	Extra-axial CSF	1.3	1.0	-0.3	2.2	0.5	4.1	3.0	3.5	-1.9	-1.4	-0.6	0.4	0.1	-1.4	1.5	-0.9	-1.9	-0.2
Global	Cerebellum and brainstem	5.7	5.5	3.6	5.5	6.6	5.5	2.7	0.9	1.0	1.5	-0.2	-0.9	-0.7	-0.3	-3.3	-1.9	-3.5	-0.9
2	Rt Parietal GM	9.5	3.6	4.6	5.1	6.4	4.5	6.0	3.9	0.1	0.7	0.1	-1.5	2.3	0.1	-4.0	-4.5	-4.0	-4.3
3	Lt Lateral ventricle	20.2	6.1	1.3	2.0	-0.2	4.8	10.9	0.3	-0.4	0.0	0.1	-0.1	-0.1	-1.0	2.0	-0.5	1.2	1.6
4	Rt Occipital GM	6.2	0.8	3.2	2.1	6.6	2.5	4.5	4.4	-0.6	1.3	1.5	-0.9	1.9	1.0	-3.5	-2.6	-2.2	-2.4
6	Lt Parietal GM	8.8	3.9	3.7	4.5	7.1	5.5	6.0	4.0	0.5	1.1	1.0	-1.3	3.2	0.0	-4.2	-4.2	-3.9	-4.5
8	Lt Occipital GM	7.0	1.6	2.9	3.7	5.2	2.8	4.2	4.1	-0.9	1.5	1.8	-0.2	0.8	0.6	-3.7	-2.9	-2.6	-1.9
9	Rt Lateral ventricle	20.4	3.1	1.3	0.7	0.3	7.1	9.2	2.2	-0.2	-0.3	0.1	-0.4	0.2	-1.2	1.1	-0.2	0.8	1.6
11	Rt Globus pallidus	0.1	-0.6	1.0	4.9	4.9	4.1	5.9	2.5	-0.2	-1.5	-0.9	-0.5	1.0	1.5	-3.1	-2.0	-3.2	-2.9
12	Lt Globus pallidus	-0.2	-0.6	-0.2	5.0	5.5	4.3	6.3	2.1	0.0	-0.5	-0.7	-0.9	1.1	1.1	-3.3	-2.0	-4.0	-3.0
14	Lt Putamen	2.2	-0.5	1.1	5.2	7.3	4.5	4.4	1.3	-0.6	-0.7	-0.2	-0.9	0.5	0.1	-3.5	-2.4	-4.2	-3.1
16	Rt Putamen	0.6	-0.2	1.5	5.1	7.1	4.7	3.5	0.9	-0.5	-1.2	-0.9	-0.7	0.3	-0.3	-3.2	-2.0	-4.0	-3.8
17	Rt Frontal WM	1.9	0.6	1.3	7.2	16.2	10.5	5.2	5.0	-0.1	0.4	-0.8	-1.5	-1.0	0.0	-1.9	-1.7	-2.1	-1.1
20	Brainstem	7.5	3.3	2.1	6.0	7.0	5.4	3.3	1.6	0.5	0.4	-0.9	-0.5	-1.6	-0.1	-2.8	-1.3	-2.8	-2.2
23	Rt Subthalamic nucleus	5.7	1.6	0.5	3.5	9.8	7.0	8.2	1.9	1.1	0.7	0.7	-0.3	0.1	0.2	-2.9	-1.9	-2.4	-2.2
29	Lt Fornix	-1.2	0.6	-1.2	4.7	3.1	2.3	-2.6	3.1	-0.9	-0.3	-0.5	0.1	-1.2	0.6	-3.4	-2.0	-2.6	-1.7

30	Lt Frontal WM	2.0	0.4	1.1	6.8	13.8	9.7	5.4	4.9	0.2	0.1	-0.9	-1.5	-1.4	0.1	-2.0	-1.7	-2.5	-0.9
33	Lt Subthalamic nucleus	2.0	0.3	1.6	2.1	7.8	6.5	6.2	2.0	1.7	1.0	-0.6	-0.8	-0.2	0.3	-2.2	-1.6	-2.7	-2.1
39	Lt Caudate	0.8	2.0	2.5	6.3	8.0	5.9	9.2	3.1	0.3	-0.4	-0.7	-1.8	1.6	-0.2	-3.1	-2.1	-3.1	-2.2
45	Rt Occipital WM	0.9	0.0	0.9	0.1	7.1	6.5	2.2	4.9	0.7	-0.2	-0.5	-1.6	-1.2	-0.8	-2.1	-1.4	-2.5	-0.6
53	Rt Caudate	4.8	2.8	3.9	5.5	6.1	6.1	7.2	3.1	0.6	-1.1	-0.6	-1.7	0.7	-0.3	-2.8	-1.6	-2.7	-2.2
57	Lt Parietal WM	0.6	0.9	1.2	4.9	12.5	9.5	4.9	4.5	1.3	0.5	-0.6	-1.9	-0.2	-0.5	-2.4	-1.8	-2.3	-1.7
59	Rt Temporal WM	2.1	0.8	1.8	3.8	10.3	8.6	3.8	3.6	-0.4	0.8	-0.8	-1.9	-0.9	-0.5	-1.8	-2.9	-2.6	-1.6
67	Lt Cerebellum	5.0	5.5	3.9	5.0	6.0	5.4	2.2	0.6	1.1	1.7	0.2	-1.0	-0.4	-0.3	-3.7	-1.7	-3.5	-0.5
73	Lt Occipital WM	0.6	-0.4	0.6	1.4	8.8	5.4	1.7	5.6	-0.6	0.2	-0.6	-2.0	-1.9	-0.4	-1.8	-1.6	-2.4	-0.1
76	Rt Cerebellum	4.6	5.6	3.4	5.0	6.0	4.7	2.7	0.8	0.9	1.6	-0.1	-0.8	-0.4	-0.4	-2.6	-2.0	-3.3	-0.5
83	Lt Temporal WM	2.4	0.9	2.3	4.2	11.1	7.6	3.4	4.2	0.2	0.8	-1.0	-2.3	-0.8	-0.5	-2.0	-2.7	-2.2	-1.1
102	Lt Thalamus	8.1	3.5	3.6	7.0	9.5	6.9	7.3	4.0	-0.6	0.0	-0.4	0.1	-0.3	-0.5	-3.3	-2.9	-2.8	-1.7
105	Rt Parietal WM	0.0	1.0	1.5	5.5	11.3	9.7	6.6	3.9	0.9	0.1	-1.6	-2.2	-1.3	-0.6	-2.4	-1.9	-2.1	-2.1
203	Rt Thalamus	6.9	3.1	3.8	6.5	9.8	6.8	7.3	5.0	-0.7	-0.1	-0.3	0.2	-0.2	-0.5	-2.5	-2.6	-2.2	-1.8
210	Lt Frontal GM	10.8	2.3	3.7	6.7	9.6	5.2	5.2	3.9	-0.2	1.5	0.8	-1.2	1.1	0.2	-4.4	-4.5	-4.2	-4.5
211	Rt Frontal GM	11.1	2.3	4.2	6.4	8.7	5.2	4.8	3.6	-0.1	1.3	0.9	-1.3	1.7	0.2	-4.5	-4.2	-3.8	-4.3
218	Lt Temporal GM	8.1	2.1	4.9	4.5	9.4	4.3	5.2	4.2	-0.1	1.9	1.0	-1.2	1.6	0.1	-4.6	-4.7	-4.4	-3.6
219	Rt Temporal GM	8.7	2.6	5.0	4.9	9.2	5.1	5.1	4.5	-0.3	1.7	1.6	-1.1	1.5	0.1	-4.3	-4.1	-3.5	-3.8
232	Third ventricle	22.5	2.5	4.1	1.2	4.9	9.1	10.4	1.3	0.1	1.9	1.7	0.2	1.7	-1.4	3.4	-0.7	1.9	2.2
233	Fourth ventricle	4.3	-0.2	0.6	0.5	-0.1	1.5	1.4	0.9	0.7	0.1	0.5	-0.7	0.4	-1.1	-0.1	-0.4	-0.1	0.1
254	Rt Fornix	-1.8	1.0	-1.0	4.1	3.8	3.3	-2.4	3.2	-0.9	-0.6	-0.8	-0.4	-1.7	0.7	-3.4	-2.6	-2.3	-1.4
255	Extracerebral CSF	-0.2	0.7	-0.4	2.2	0.5	3.7	2.4	3.5	-1.9	-1.4	-0.6	0.4	0.0	-1.3	1.4	-0.9	-2.0	-0.3

Abbreviation: CSF, cerebral spinal fluid system; F, female; GM, gray matter; GS, Gorlin syndrome; M, male; NC, Neurotypical control; PHTS, PTEN hamartoma tumor syndrome; RTT, Rett syndrome; MS, Malan syndrome; WM, white matter.
