# Supplementary material for: A Brain Morphometry Study with Across-Site Harmonization Using a ComBat-Generalized Additive Model in Children and Adolescents
Source: Diagnostics (Basel). 2023 Aug 27;13(17):2774. doi: 10.3390/diagnostics13172774 (PMC10487204; doi:10.3390/diagnostics13172774)
Supplement: Supplementary file 1 [file diagnostics-13-02774-s001.zip › BASH-NC Table S3.pdf]

Table S3: Laterality index of volumetric measurements of each additional case

Case	1	2	3	4	5	6	7	8	9	10	11	12	13	14	15	16	17	18
Sex	Male	Male	Male	Male	Male	Male	Male	Male	Female	Female	Female	Female	Female	Female	Female	Female	Female	Female
Age at scan (years)	9.6	13.3	13.7	10.3	9.1	9.4	8.6	14	8.8	15.2	13.8	6.7	6.6	7.3	11.1	9.6	15.4	8.3
Disorder	GS	GS	GS	MS	PHTS	PHTS	PHTS	PHTS	NC	NC	NC	RTT	RTT	RTT	RTT	RTT	RTT	RTT
Scan-site	CUH	CUH	CUH	CUH	BCH	BCH	BCH	BCH	CHBC	CHBC	CHBC	BCH	BCH	BCH	BCH	CHBC	CHBC	CHBC
**Description**																		
Frontal GM	-0.7	-0.4	-2.1	0.6	1.9	-0.1	1.2	0.8	-0.7	0.2	-0.4	0.3	-2.5	-0.1	0	-4.3	-3.7	-2.6
Temporal GM	-2.6	-3.5	-1.8	-2.2	-0.7	-3.3	-1.2	-2.3	-0.1	-0.3	-0.3	-1.2	-0.3	-0.8	-5.2	-6.8	-6.7	-0.4
Parietal GM	-1.5	1.9	-2.7	-1.7	2.7	4.3	0.6	1.3	2.5	2.7	4.2	1.6	4.6	0.0	-3.5	1.3	1.0	-4.2
Occipital GM	2.1	3.1	-2.0	5.9	-5.5	0.7	-3.5	-1.8	-2.9	0.2	-6.8	3.5	-7.8	-3.8	0.4	-2.2	-3.9	4.9
Frontal WM	1.0	-1.3	-0.7	0.3	-3.0	-0.3	0.7	-0.2	1.9	-0.5	1.4	-0.7	-2.7	1.0	-0.8	0.3	-2.7	1.0
Temporal WM	0.4	0.3	2.0	0.6	1.0	-3.7	-2.8	1.7	4.0	0.1	-1.2	-2.8	1.0	-0.2	-1.5	1.7	3.6	3.9
Parietal WM	4.1	-0.3	-1.4	0.3	6.1	2.6	-4.6	2.6	2.7	3.1	5.4	1.6	7.7	0.9	0.1	1.7	-2.5	2.1
Occipital WM	-3.0	-3.6	-3.4	6.6	2.0	-7.0	-3.3	0.6	-9.8	1.7	-4.0	-2.9	-6.4	2.8	3.6	-1.0	0.4	3.7
Globus pallidus	0.5	1.7	-4.4	1.0	2.5	1.6	2.3	-0.2	3.0	7.0	2.7	-0.3	2.5	0.4	-1.9	0.8	-4.3	0.1
Putamen	4.8	-3.2	-3.2	-1.2	-0.9	-2.0	1.7	0.6	-2.4	1.0	-0.9	-2.8	-0.3	0.6	-4.7	-4.6	-3.1	2.1
Fornix	4.8	-0.6	0.3	4.6	0.1	-1.4	-1.2	0.8	0.9	3.6	1.5	4.5	3.9	1.4	1.0	6.6	-0.5	-1.8
Caudate	-13.8	-3.3	-5.6	2.3	5.0	-0.9	5.8	0.2	-0.4	3.9	-2.0	-0.3	4.7	1.1	-2.2	-3.7	-2.5	0.6
Thalamus	2.7	1.6	0.0	1.3	-0.8	0.2	0.3	-2.4	0.5	0.8	-0.5	0.2	-0.3	0.5	-3.4	-1.0	-2.2	0.4
Subthalamic nucleus	-14.1	-8.4	6.4	-5.7	-3.9	-0.2	-8.4	0.7	2.3	2.7	1.5	-4.1	-3.1	0.1	6.4	2.3	-1.8	1.8
Lateral ventricle	7.0	**32.9**	7.2	**24.5**	-12.7	-8.8	8.0	**-26.3**	0.6	12.4	8.4	14.5	-3.0	18.4	14.2	-5.6	9.7	6.3
Cerebellum	1.2	-0.3	1.5	0.0	-0.1	1.9	-1.3	-1.0	1.4	0.2	-0.1	-0.6	0.2	0.6	-6.0	1.4	-0.6	0.0

Bold indicates values of over absolute 20, indicating asymmetry. Abbreviation: GM, gray matter; GS, Gorlin syndrome; NC, Neurotypical control; PHTS, PTEN hamartoma tumor syndrome; RTT, Rett syndrome; MS, Malan syndrome; WM, white matter.
